# Immunotherapy of acute leukemia by chimeric antigen receptor-modified lymphocytes using an improved *Sleeping Beauty* transposon platform

**DOI:** 10.18632/oncotarget.9955

**Published:** 2016-06-13

**Authors:** Chiara F. Magnani, Nice Turazzi, Fabrizio Benedicenti, Andrea Calabria, Erika Tenderini, Sarah Tettamanti, Greta M.P. Giordano Attianese, Laurence J.N. Cooper, Alessandro Aiuti, Eugenio Montini, Andrea Biondi, Ettore Biagi

**Affiliations:** ^1^ Tettamanti Research Center, Department of Pediatrics, University of Milano-Bicocca, San Gerardo Hospital/Fondazione MBBM, Monza, Italy; ^2^ San Raffaele Telethon Institute for Gene Therapy (HSR-TIGET), Milan, Italy; ^3^ University of Texas MD Anderson Cancer Center, Houston, Texas, USA

**Keywords:** chimeric antigen receptor, Sleeping Beauty transposon, cytokine-induced killer cells, acute lymphoblastic leukemia, acute myelogenous leukemia

## Abstract

Chimeric antigen receptor (CAR)-modified T-cell adoptive immunotherapy is a remarkable therapeutic option proven effective in the treatment of hematological malignancies. In order to optimize cell manufacturing, we sought to develop a novel clinical-grade protocol to obtain CAR-modified cytokine-induced killer cells (CIKs) using the Sleeping Beauty (SB) transposon system. Administration of irradiated PBMCs overcame cell death of stimulating cells induced by non-viral transfection, enabling robust gene transfer together with efficient T-cell expansion. Upon single stimulation, we reached an average of 60% expression of CD123- and CD19- specific 3rd generation CARs (CD28/OX40/TCRzeta). Furthermore, modified cells displayed persistence of cell subsets with memory phenotype, specific and effective lytic activity against leukemic cell lines and primary blasts, cytokine secretion, and proliferation. Adoptive transfer of CD123.CAR or CD19.CAR lymphocytes led to a significant anti-tumor response against acute myelogenous leukemia (AML) and acute lymphoblastic leukemia (ALL) disseminated diseases in NSG mice. Notably, we found no evidence of integration enrichment near cancer genes and transposase expression at the end of the differentiation. Taken all together, our findings describe a novel donor-derived non-viral CAR approach that may widen the repertoire of available methods for T cell-based immunotherapy.

## INTRODUCTION

Adoptive transfer of chimeric antigen receptor (CAR)-modified T lymphocytes has recently been proposed as advanced treatment for relapsed and refractory chronic lymphocytic leukemia, acute lymphoblastic leukemia (ALL), CD19-positive lymphomas and multiple myeloma. [1–8] Developed to improve the graft-versus-leukemia effect, CAR molecules are designed by fusing the antigen-binding domain of a monoclonal antibody in the form of a single-chain fragment variable (scFv) with a signal transduction domain, usually the z chain of the TCR and costimulatory molecules, in a unique artificial receptor. [9] In this regard, CD19-directed CAR treatment of patients resulted in persistence of immunological memory, trafficking to the tumor sites, and non-HLA-restricted anti-tumor activity, which led to tumor regression and, in most of the patients, complete remission. [1–4, 6]

Concerning the aspect of *ex vivo* T-cell modification, in the past two decades, viral vectors have constituted a valuable tool for successful gene therapy thanks to their efficacy in mediating stable gene transfer into primary cells with standardized good manufacturing practice (GMP)-grade processes [10, 11] and overall safety in modifying differentiated immune cells. [12] In parallel, non-viral gene transfer methods have recently been developed with the goal of overcoming high manufacturing costs, regulatory hurdles and scale-up complexities, which have limited so far the range of application of CAR-based immunotherapy with respect to other easier approaches such as monoclonal antibodies (mAbs). [13] However, commonly available non-viral methods are based on transient transfection by mRNA electroporation [14, 15] or stable, integrative methods that have limited transfection efficiency. In this context, the *Sleeping Beauty* (SB) transposon plasmid system [16] is quite inexpensive and easy to produce and purify. Furthermore, SB appears to be less immunogenic than viral vectors and, because it integrates randomly into the host genome, [17, 18] it retains a safer pattern compared to gamma retroviral vectors, which have the tendency to target gene promoters, thereby having an increased probability to induce aberrant gene expression. [19, 20] Thus, SB has been used in combination with electroporation for gene transfer in human primary T cells with the limitation of relatively low transfection efficiency. [21] Using the SB method, Singh *et al.* have successfully generated CD19-redirected CAR-modified T cells for Phase I and II clinical trials. [22] In order to obtain a consistent amount of CAR^+^ T cells, the authors expanded and, simultaneously, selected effector cells by repetitive stimulation with CD19^+^ artificial APC. [23]

With regard to the development of CAR therapies using cytokine-induced killer (CIK) -cell cultures, [24] effector lymphocytes with acquired NK-like cytotoxicity are usually generated by culturing PBMCs in the presence of IFN-γ, IL-2, and anti-CD3 mAbs. This cell population expresses T-cell markers (> 97% are CD3^+^) and it is enriched in highly cytotoxic CD3+CD56+ cells. In the context of leukemia immunotherapy, we have previously shown that anti-CD19 and anti-CD123 CARs redirected the activity of CIK cells against primary ALL and AML blasts, respectively. [25–27] The advantage of choosing donor-derived CIK-cell cultures stems from the fact that these cells display a non-HLA-restricted cytotoxicity [24] along with minimal alloreactivity. [28] Furthermore, it has been shown that an easy protocol could promote their rapid expansion *ex vivo* under validated pharmaceutical GMP conditions. [29] However, to our knowledge, none of the currently published non-viral methods has reached significant efficiency to be applied to easy-to-translate T-cell protocols. [23, 30–32] Here, we describe the development of a unique non-viral clinical-grade immunotherapy approach for acute leukemias. We were able to achieve stable and efficient CAR expression and, concomitantly, boost cell expansion while minimizing cell manipulation and preserving phenotype, viability, and effector functions of the redirected cells. In addition, we performed molecular analysis of SB-engineered CIK cells by high-throughput genomic integration site retrieval, bioinformatics, and transposase expression analysis.

## RESULTS

### Transfection of primary T-cell precursors and CIK-cell differentiation by SB

**Figure 1:**
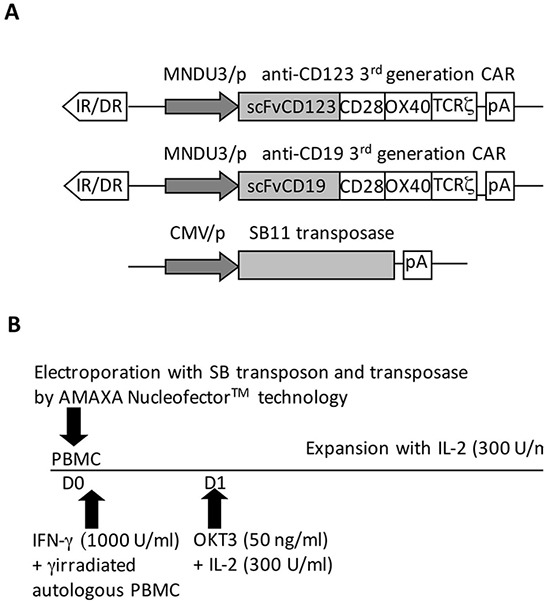
Modification and expansion of mononuclear precursors by the SB system **A.** The diagram of the SB transposon and transposase constructs used in this study, encoding for CD123.CAR (upper panel), CD19.CAR (middle panel), and transposase (lower panel), is shown - IR/DR, SB inverted repeats/directed repeats; MNDU3/p, the constitutive promoter from the U3 region of the MND retrovirus; scFv, single chain fragment variable; pA, polyadenylation signal from bovine growth hormone; CMV/p, CMV promoter. **B.** The expansion and modification protocol used in this study is shown. PBMC from HDs were nucleofected at D0 with the transposon and transposase constructs using Amaxa Nucleofector^TM^ technology. According to standard differentiation protocol, IFN-γ was added after nucleofection, whereas simultaneous addition of γ-irradiated autologous PBMCs was performed to re-establish the myeloid fraction of PBMCs impaired by the modification procedure. At day 1, the expansion protocol was started with OKT3 and IL-2 and the cells were cultured in the presence of IL-2 until day 21.

**Figure 2:**
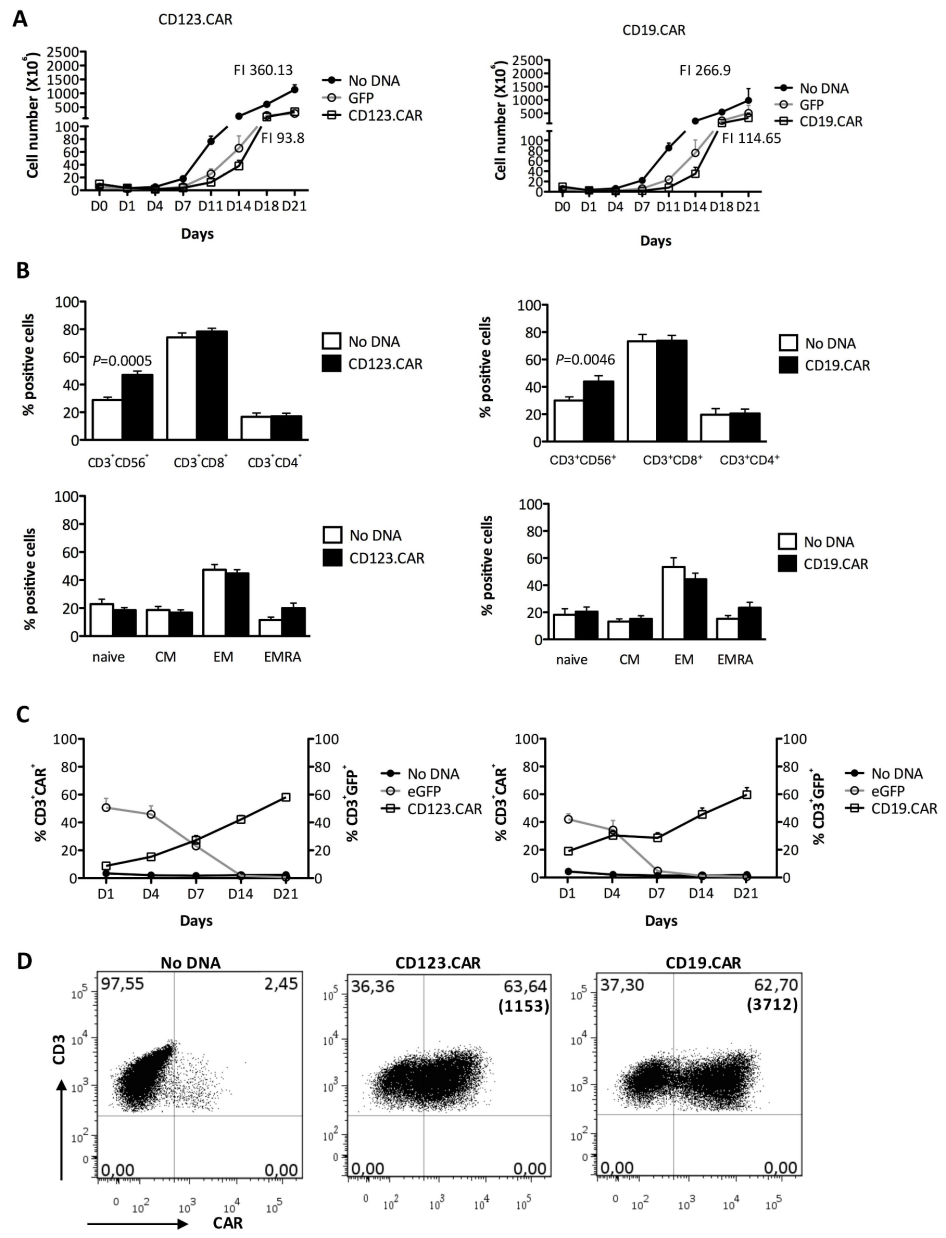
Expansion, phenotype, and transgene expression of SB-modified CIK cells **A.** Proliferation of cells nucleofected in the absence of DNA, with GFP, and with SB encoding CD123.CAR (left panel) or CD19.CAR (right panel) were followed overtime by cell count. Fold increase (FI, total cell number at day 21 / cell count at day 1) is indicated. **B.** CD56/CD8/CD4, Central memory (CM)/Effector memory (EM)/Effector memory RA (EMRA) phenotype at day 21. **C.** Modification was determined overtime by flow-cytometry. **A-C.** Mean±SEM were as follows: No DNA, CD123.CAR (n=13) and GFP (n=8); No DNA, CD19.CAR (n=8) and GFP (n=4). **D.** CAR expression was determined at day 21 of differentiation in one representative donor. Numbers represent the percentages of positive cells and MFI in bracket. P-values of the Paired t test are indicated.

First, we developed an optimized clinical-grade protocol to generate CIK-cell cultures expressing two distinct 3^rd^ generation CARs (Figure 1). Nucleofection of PBMCs in the presence of SB plasmids caused consistent loss of the CD11c^+^ myeloid dendritic cells (DCs) and CD14^+^ monocytes and cell mortality. After nucleofection, the addition of γ-irradiated autologous PBMCs, as source of antigen-presenting cells (APC), partially restored the above mentioned loss of DCs and monocytes. This strategy, together with the concomitant stimulation by OKT3, rescued the impaired T-cell expansion observed using various nucleofection programs while preserving high CAR expression (Supplementary Figure S1A-S1C). Cell survival rates at 24 hours reached a value of 52.6% (±6.2, n=13) for CD123.CAR and 45.0% (±8.4, n=7) for CD19.CAR (Supplementary Figure S1D-S1E). Both CD123.CAR and CD19.CAR CIK cells showed efficient expansion without requiring any additional stimulation reaching, after 3 weeks, a 93.8±26.3 and 114.7±63.8 fold increase, with an average of 121 and 161 fold expansion of CD3^+^ T cells (Figure 2A).

At the end of the differentiation, cell viability was well-preserved with an average of 75.6% in CD123.CAR and 80% in CD19.CAR (Supplementary Figure S2A-S2B). Our protocol minimally altered the phenotype of the CIK-cell product, leading to a slightly higher, albeit significant, proportion of CD3^+^CD56^+^ (43.4%±3.5 in CD123.CAR *vs.* 26.4±2.3 in control cells (No DNA); 43.9%±4.3 in CD19.CAR *vs.* 30.0±2.6 in No DNA) (Figure 2B). Nucleofection average efficiency, as assessed by GFP expression levels at 24 hours, was 50.7% (±6.5, n=11) in CD123.CAR and 42.0% (±4.0, n=4) in CD19.CAR. The expression levels of CD123.CAR and CD19.CAR were stable in CIK cells and after 21 days reached values of 58.1%±2.7 (n=13) and 59.7%±5.1 (n=8), respectively (Figure 2C-2D), which were slightly higher than the transposon encoding GFP (SB GFP, Supplementary Figure S2C). GFP alone and SB GFP differed in the kinetic of the expression pattern. CAR molecules were stably expressed by each cell subset (i.e. CD3^+^CD56^+^, CD3^+^CD8^+^, and CD3^+^CD4^+^ cells) and found to be present in cells at all stages of differentiation and memory (Supplementary Figure S2D). Taken together, these data demonstrate that replacing impaired nucleofected stimulating cells with precursor PBMCs results in optimal expansion of CAR^+^ CIK cells.

### Redirected activities of CD123. and CD19. CAR CIK cells against AML and ALL blasts

**Figure 3:**
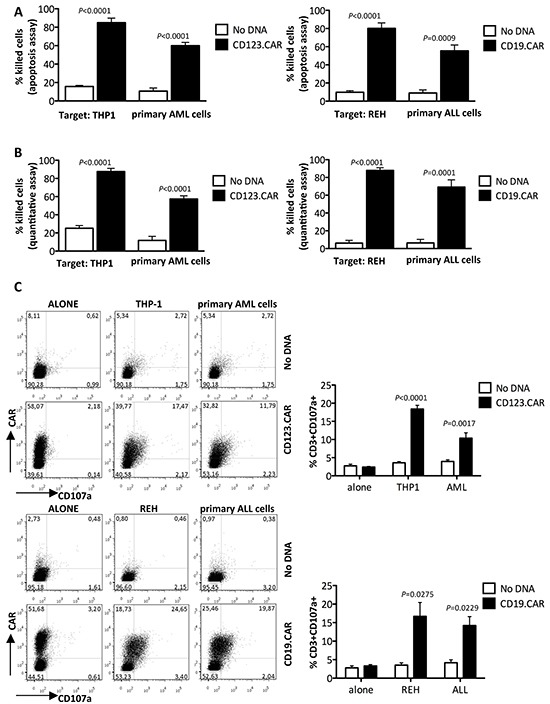
CD123.CAR and CD19.CAR redirect CIK-cell activity against CD123+ and CD19+ cells **A.** Cytotoxic activity of modified CIK cells against target cells was determined by apoptosis detection assay. The E:T ratio was 5:1. Mean±SEM of THP-1 (n=6), REH (n=8), primary AML (n=10), and ALL cells (n=6) are indicated. **B.** Cytotoxic activity by quantitative detection was determined at an E:T ratio of 5:1. Mean±SEM of THP-1 and REH (n=7) and of primary AML (n=8) and ALL (n=7) cells are shown. **C.** CD107a expression was measured in CD123.CAR- or CD19.CAR- modified CIK cells co-cultured with target cells at an E:T ratio of 1:1. Representative results from one out of 9 donors for THP-1, 7 primary AML and 5 REH and ALL cells. Numbers represent the percentages of CD107a+ cells. Mean±SEM values are plotted alongside. P-values of the Paired t test are shown.

An efficient lysis of AML THP-1 cell line (85.0%±4.9) and primary blasts (60.0%±3.6) by SB-modified CD123.CAR CIK cells was observed at the 4-hour time point at an effector target ratio of 5:1. Similar redirected cell killing was observed for CD19.CAR CIK cells incubated with ALL REH cell line (80.0%±6.0) and primary blasts (56.8%±7.7). In contrast to that, we did not observe significant cell killing by control CIK cells (No DNA) (Figure 3A). Both CD123 and CD19 antigen expression on target cells were assessed by flow cytometry (Table 1). We obtained similar results when we measured cell killing using a quantitative cytotoxic assay (Figure 3B). In addition, when CD123.CAR CIK cells and CD19.CAR CIK cells were cocultured with leukemic blasts, they showed specific cytotoxic degranulation according to CD107a expression. Notably, cytotoxic degranulation was associated with CAR expression, further indicating specific target recognition by CAR and subsequent cell killing (Figure 3C).

**Table 1:** Patients' characteristics^a^

	Age	Diagnosis	Subtype^b^	%CD33+	%CD123+	Karyotype and Gene Mutations	Prognosis
UPN1	8 y^c^	AML	M4	50.0	43.7	46, XX, inv(16)(p13q22)[16]/46, XX [4];normal FLT3-ITD^e^, normal NPM1a	SR^f^
UPN2	13y	AML	M2	86.0	71.3	46,XX,t(8;21)(q22;q22)[20];normal FLT3-ITD	SR
UPN3	4y	AML	M5a	86.0	95.5	47-48,XX,del(2)(p12),del(5)(p12),?t(6;7)(q21;q32),t(9;?)(q34;?),−11,del(12)(p11),+19,+4markers [cp9]/46,XX [3];normal FLT3-ITD, normal NPM1at(10;11) positive by RT-PCR	HR^g^
UPN4	16y	AML	M2	85.0	56.6	45,XY,t(8;21)(q22;q22)[6]/46,XY [6]	SR
UPN5	9y	AML	M0	95.0	99.0	46,XX [9]normal FLT3-ITD, normal NPM1a	HR
UPN6	12y	AML	M1	48.0	93.5	46,XY [25]NPM1+; FLT3 D835+	SR

	Age	Diagnosis	Subtype	%CD10+	%CD19+	Karyotype and Gene Mutations	Prognosis
UPN7	8y	ALL	BALL-IV	90.6	91.0	46,XY,t(8;14)(q24;q32)[20]	HR
UPN8	4y	ALL	BALL-III	96.4	22.3	47,XX,+21c [14] (Down)	HR
UPN9	8m^d^	ALL	BALL-I	5.6	70.2	46,XY [14]	SR

a Karyotype defined as International Standing Committee on Human Cytogenetic Nomenclature (ISCN) 2013;

b ALL subtype from classification EGIL [52];

c y= years;

d m= month;

e ITD= internal tandem duplication;

f SR= standard risk;

g HR= high risk;

[ ], number of metaphases analyzed.

**Figure 4:**
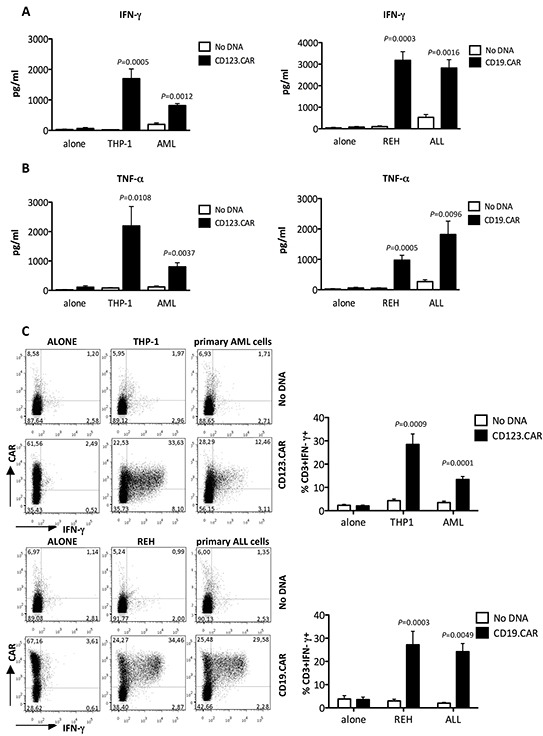
Specific cytokine production of CD123.CAR and CD19.CAR CIK cells **A-B.** The IFN-γ (A) and TNF-α (B) production was determined by ELISA upon stimulation of CD123.CAR CIK cells with THP1 and primary AML cells and CD19.CAR CIK cells with REH or primary ALL cells. Mean±SEM of THP-1 (n=10) and REH (n=8), and of primary AML and ALL cells (n=7) are shown. **C.** In parallel, IFN-γ expression was determined by intracytoplasmic staining. Representative results for one donor out of 9, for CD123.CAR, and 8, for CD19.CAR, are shown. Numbers represent the percentages of positive cells. Mean±SEM values are plotted alongside. P-values of the Paired t test are indicated.

In addition to that, we evaluated specific pro-inflammatory cytokine secretion of SB-modified CIK cells. Both CD123.CAR and CD19.CAR CIK cells exposed to THP-1 and AML primary cells or REH and ALL primary cells, respectively, released a significant higher amount of IFN-γ and TNF-α compared to No DNA CIK cells (Figure 4A-4B). CD123.CAR CIK cells produced both IFN-γ (28.5%±4.5 with THP-1, 13.4%±1.3 with primary AML, n=9) and IL-2 (10.1%±2.0 with THP-1; 5.8%±1.1 with primary AML, n=9), assessed by intracytoplasmic staining (Figure 4C and Supplementary Figure S3). Likewise, CD19.CAR CIK cells produced both IFN-γ (27.2%±5.8 with REH; 24.2%±3.5 with primary ALL) and IL-2 (10.1%± 1.2 with REH; 10.6%±1.6 with primary ALL). The response was restricted to CAR^+^ CIK cells, indicating that cytokine secretion is stimulated upon specific CAR-triggering by the antigen expressed on leukemic cells.

We next directly compared our platform with the already established methods of conventional T-cell modification by SB. [31, 32] Our data showed expansion of CIK cells at higher rate compared to OKT3- and beads- activated T cells with a fold increase of 35.6±7.1 versus 13.4±4.3 and 7.2±3.9, respectively. Addition of γ-irradiated autologous PBMCs increased both OKT3- and beads- activated T-cell expansion (Supplementary Figure S4A-S4B). CAR expression was similar in all conditions with the exception of the lower expressing beads-activated T cells (Supplementary Figure S4C-S4D). CIK cells were slightly superior in cytotoxicity (Supplementary Figure S4E-S4F) and cytokine secretion ability (Supplementary Figure S4G-S4H). The observed difference of CAR expression in beads-activated T cells and of cytotoxicity in OKT3-activated T cells compared with CIK cells was restored by addition of γ-irradiated PBMCs.

### SB-engineered CIK cells proliferate upon CAR-specific stimulation

**Figure 5:**
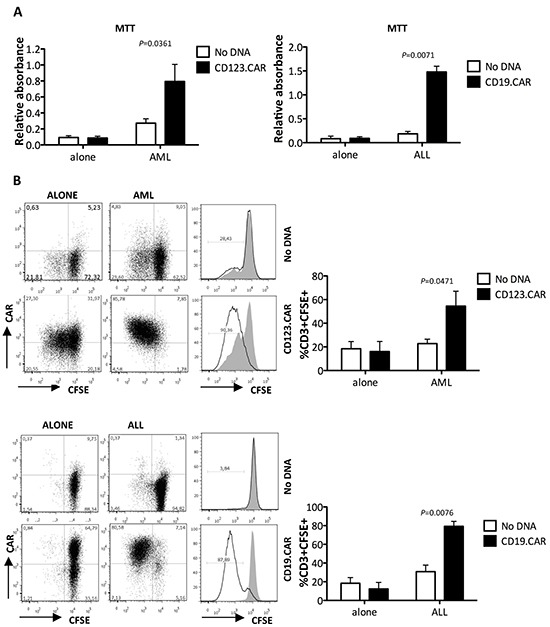
CD123.CAR and CD19.CAR CIK cells proliferate in response to CD123+ and CD19+ blasts **A.** Proliferation of No DNA, CD123.CAR and CD19.CAR CIK cells after stimulation with AML or ALL cell lines was determined by MTT assay. Mean±SEM of CD123.CAR (n=8) and CD19.CAR (n=5) are shown. **B.** Proliferation was determined by CFSE standard assay. Representative results from one out of 5 donors are shown. Numbers represent the percentages of positive cells. Mean±SEM values are plotted alongside. P-values of the Paired t test are indicated.

We then evaluated whether CD123.CAR and CD19.CAR constructs containing a 3^rd^ generation signaling domain could lead to specific proliferation. For this purpose, CD123.CAR and CD19.CAR CIK cells were cocultured with THP-1 and REH cell lines, respectively in the absence of rhIL-2. CD123.CAR and CD19.CAR CIK cells proliferated in the presence of AML and ALL cells, respectively, as determined by MTT assay. We observed a limited non specific proliferation of No DNA CIK cells after incubation with THP-1, but not with REH (Figure 5A). In order to better evaluate specific proliferation, CIK cells were co-stained with CFSE [33] and CAR-specific mAb. Antigen-triggered CAR activation led to specific proliferation of both CD123.CAR (54.5%±12.6 *vs.* 22.8%± 3.7 of No DNA; p=0.0471, n=5) and CD19.CAR CIK cells (79.3%±5.3 *vs.* 30.8%± 7.0; p=0.0076, n=5). In particular, the proliferating CFSE^low^ fraction cells were mainly CAR^+^, suggesting specific activation and selection of modified CIK cells upon encounter with cancer cells (Figure 5B).

### SB-engineered CIK cells exert anti-tumor responses *in vivo*

**Figure 6:**
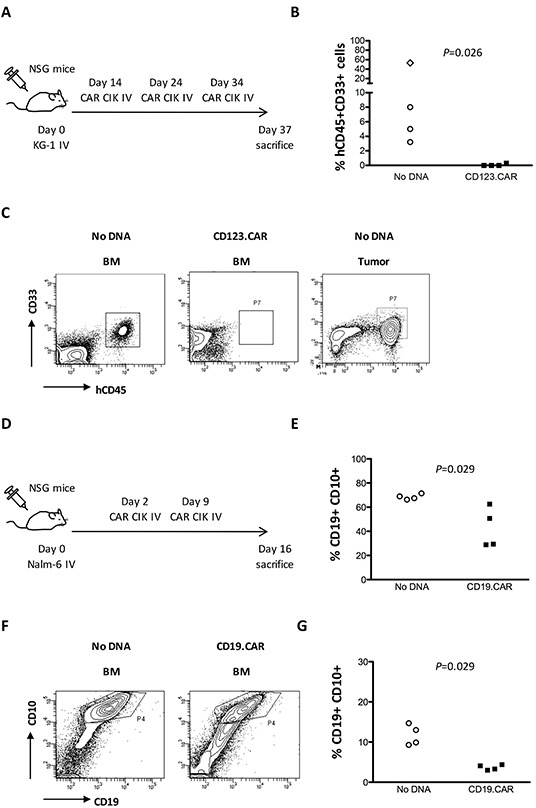
*In vivo* anti-tumor activity of CD123.CAR and CD19.CAR CIK cells **A.** Schematic representation of the AML experiment. Engraftment was measured based on the presence of mouse CD45^−^ human CD45^dim^ CD33^+^ cells by BM puncture. Infusion of CIK cells was performed on days 14, 24 and 34 after transplantation. Mice were sacrificed after 37 days. **B-C.** KG-1 engraftment in the BM or in extramedullary tumors (rhombus). Each dot represents a mouse. Representative results from one CD123.CAR donor and one No DNA donor are shown. **D.** Schematic representation of the ALL experiment. Engraftment was measured based on the presence of mouse CD45^−^ CD10^+^ CD19^+^ cells by BM puncture. Infusion of CIK cells was performed on days 2 and 9 after transplantation. **E-G.** Mice were sacrificed after 16 days. Nalm-6 engraftment in the BM (E-F) and spleen (G). Each dot represents a mouse. Representative results from one CD19.CAR donor and one No DNA donor are shown. P-values of the Mann Whitney test are indicated.

We next evaluated the *in vivo* efficacy of CD123.CAR and CD19.CAR CIK cells against AML and ALL, respectively. For this purpose, we injected AML KG-1 or ALL Nalm-6 cells into the tail vein of immunodeficient NOD-SCID-γchain−/− (NSG) mice. Mice were grafted with KG-1 cells and, after 14 days, received an intravenous infusion of 1×10^7^ CD123.CAR or No DNA CIK cells from the same donor every 10 days, as previously reported [27] (Figure 6A). KG-1 cells engrafted either as disseminated leukemia or extramedullary tumor in animals treated with No DNA CIK cells. Conversely, treatment with CD123.CAR CIK cells eradicated KG-1 cells in the BM and significantly inhibited tumor growth as compared to No DNA CIK-treated mice (Figure 6B-6C). No extramedullary tumor was found in mice treated with CD123.CAR CIK cells.

In the ALL model, NSG mice were grafted with Nalm-6 cells and, subsequently, infused with 1×10^7^ CD19.CAR or No DNA CIK cells from the same donor at day +2 and +9 (Figure 6D). BM and spleen organs were collected and a significant reduction of tumor growth by CD19.CAR CIK cells was observed compared to No DNA CIK-treated mice (Figure 6E-6G). Collectively, these results indicate a potential therapeutic effect of multiple injections of SB-engineered CAR CIK cells in the absence of simultaneous administration of rhIL-2.

### Safety and efficacy assessment in SB marked CIK cells

**Figure 7:**
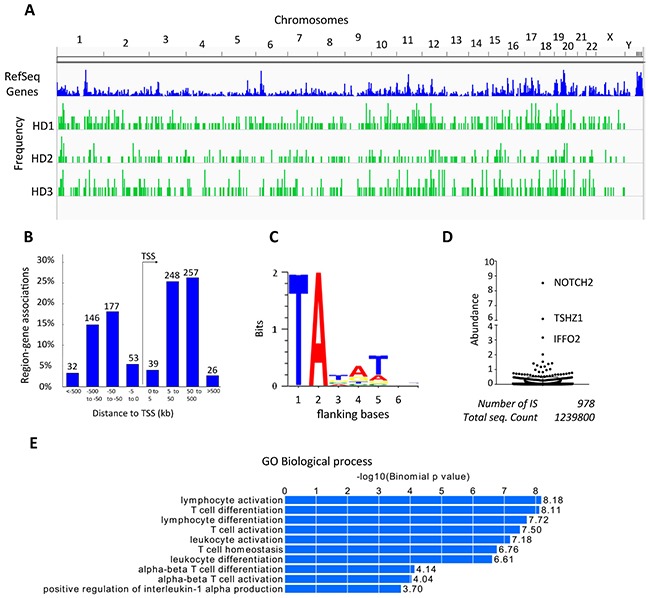
Integration site analysis in transduced CIK-cell cultures **A.** Graphic representation of the distribution of integrations at chromosome level in the genome of each HDs. **B.** Frequency distribution of SB integrations around the Transcription Start Site (TSS) (intervals in Kb, x-axis) of the nearest target gene (in %, y-axis). The number of integrations mapping in each genomic interval is indicated above each bar. **C.** logo-plot representation of the bases flanking the SB integration sites - the positions of the bases after the SB integration site are indicated in the X-axis - showing the characteristic TA motif present at each SB integration. **D.** Relative clonal abundance of clones harboring specific ISs; y-axis, % of sequencing reads with respect to the total sequencing reads found for HD1, HD2 and HD3 pooled together. The name of the nearest target gene is indicated for clones with abundance >3%. **E.** Overrepresented gene classes of the Gene Ontology (GO) Biological process targeted by SB integrations.

Next, we assessed the genomic distribution of SB integration sites (IS) by linear amplification-mediated (LAM)-PCR from the genomic DNA of CD123.CAR CIK cells. Spreadex gel electrophoresis of the LAM-PCR products showed a smeared pattern (Supplementary Figure S5), suggesting a polyclonal repertoire consistent with the TCR-Vβ analysis (Supplementary Figure S6A-S6B). PCR products were subject to Illumina MiSeq next-generation sequencing, and the IS were mapped on the human genome using a previously described bioinformatics pipeline. [34, 35] This approach allowed us to retrieve 1,239,800 sequencing reads with valid vector/cellular genome junctions, corresponding to 978 of unique IS (473, 212 and 293 in healthy donor (HD) 1, 2, 3, respectively) (Supplementary Table S1). Considering that each IS has a unique genetic mark which allows the identification and tracking of a cell clone and its progeny among vector-marked cells, the high number of IS retrieved further supports the presence of a polyclonal repertoire. In accordance with De Jong J. et *al*., [36] and Gogol-Doring et *al*., [37] the SB IS were randomly distributed along the genome without preferences for gene dense regions and characterized by a low tendency to target gene promoters (Figure 7A-7B). Moreover, we were able to identify the canonical AT-rich conserved consensus at the genomic TA dinucleotides flanking the IS [T A T A/G T, Figure 7C]. [18] The clonal abundance was estimated as the relative percentage of sequence counts representing each IS with respect to the total of sequences retrieved in the analysis. No signs of dominance of individual clones emerged from this analysis (Figure 7D and Supplementary Table S2). Finally, we sought to determine whether SB integrations in specific gene classes or genomic locations (i.e. common insertion sites, CIS) were significantly enriched, which would have indicated a selective advantage conferred by this type of integrations. Gene ontology-based overrepresentation analysis using GREAT online software (http://bejerano.stanford.edu/great/public/html/) [38] showed a significant enrichment of genes expressed in T cells (Figure 7E), in agreement with the known moderate preference of SB transposons to integrate within expressed genes. [36, 37] CIS significance analysis was performed using Montecarlo simulations considering only CIS constituted by at least 4 IS contained in a window of 100Kb. We could not identify any CIS using this method (Supplementary Table S3).

**Figure 8:**
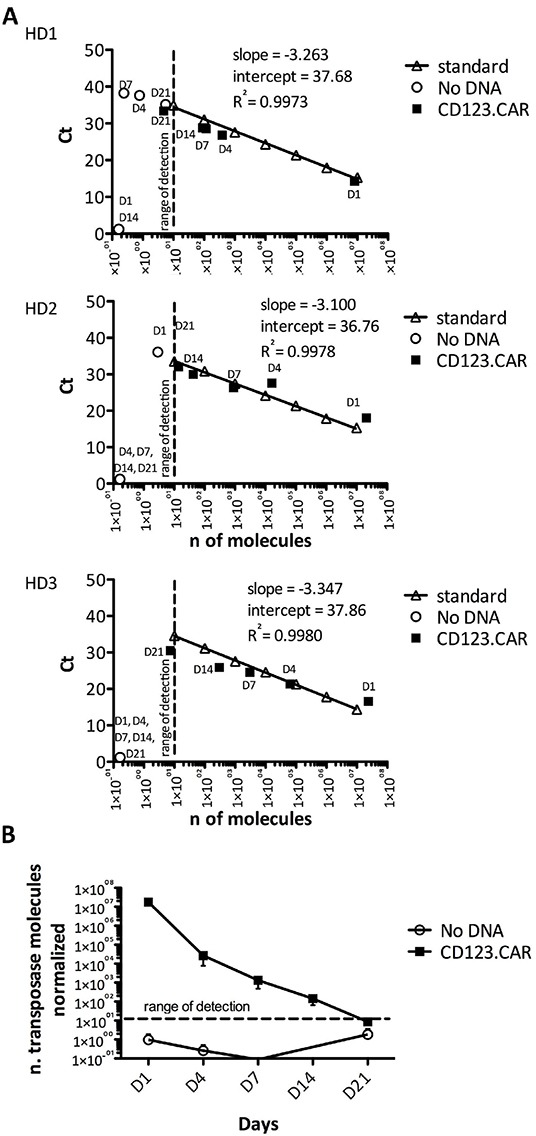
Transposase clearance in transduced CIK cells **A.** Expression analysis of transposase by quantitative real time PCR (Q-RT-PCR) in No DNA control cells and CD123.CAR cells on days 1, 4, 7, 14, 21 during differentiation (n=3). Slope, coefficient of determination (R2), and intercept of the standard curve are shown. **B.** Evaluation of the transposon expression in CIK cell cultures over time by Q-RT-PCR, as number of transposase molecules normalized to 10^4^ GUS copies. Mean±SEM from 3 donors are shown.

It is established that the SB11X transposase-expressing plasmid, although at low frequency, can integrate randomly into the host genome and express the transposase, which could potentially lead to remobilization of the transposon in other genomic compartments. To evaluate the kinetics of SB11X transposase expression during CIK-cell culture, which could jeopardize the stability of the genomic content of the final cell product, we developed a quantitative RT-PCR assay. The slope of the standard curves was between 3.1 and 3.4 with a correlation coefficient >0.99 (Figure 8A). The quantities of transposase were detected at day 1, 4, 7, 14, and 21 in CIK cells and normalized to 10^5^ GUS molecules (Figure 8B). The expression of transposase enzyme after nucleofection, which was of about 10^7^ normalized molecules, was gradually lost overtime as the plasmids were progressively degraded and diluted by cell proliferation and fell below the limit of detection in the final cellular products (Figure 8A-8B).

## DISCUSSION

In this study, we provide evidence of an innovative GMP-grade SB transposon platform which can efficiently modify and expand CAR^+^ lymphocytes. Both CD123.CAR and CD19.CAR CIK cells exhibited a fully competent T-cell response characterized by specific killing, cytokine secretion and proliferation. Furthermore, SB-engineered CIK cells showed anti-tumor activity *in vivo*. Overall, gene transfer by SB displayed a safe pattern of integrations into the host genome and did not lead to transposase expression in the final T-cell product.

Transposon gene transfer has been previously carried out in primary human T cells. However, the significant cell damage induced by electroporation due to the strong magnetic field and high quantities of DNA severely impaired the survival of modified T cells. To overcome this limitation, several groups reported that the combined use of repetitive stimulations and specific selection using microbeads, [39] cell sorting, [21, 31, 32] or artificial APC [23] could generate higher numbers of modified T cells. In our hands, electroporation of PBMCs with SB plasmids led to complete depletion of DC and monocytes resulting in poor expansion of modified cells upon stimulation with anti-CD3 mAb. In order to re-establish a physiological T cell-APC contact, which then would enhance cell survival and stimulation rates, we added irradiated PBMCs, derived from the same source, after nucleofection. To our knowledge, this is the first approach demonstrating that high efficiency could be achieved by non-viral modification of primary human T cells with limited manipulation and stimulation requirements.

The kinetics of CAR expression in modified CIK cells raises the question whether CAR^+^ cells are selected during the differentiation or impaired in transgene expression after nucleofection. The polyclonal pattern observed in the TCR-Vβ and integration analysis indicates that modified CIK cells do not undergo a selection process of specific clones. Furthermore, the delayed expansion, with respect to No DNA CIK cells, suggests that the toxicity associated with nucleofection does indeed inhibit the cellular machinery. However, the lower transgene expression in the SB-GFP condition raises the possibility that specific signaling by CD123^+^ and CD19^+^ PBMC subsets could stimulate CAR-transduced cells, thus explaining the CAR expression kinetic.

The rationale for using CIK over standard CAR T cells comes from our clinical experience clearly demonstrating that repeated infusions of CIK cells in patients are safe and well-tolerated. [29] The possibility of using safe donor-derived cells is of particular relevance in patients who are lymphodepleted. [40] Up to now, nearly 3,000 patients, enrolled in 45 clinical trials investigating 22 different tumors in 25 years, have been treated with CIK cells. [41] Here, we provide evidence that CIK cells obtained through our customized non-viral method are characterized by high viability and phenotypic identity. Furthermore, the final cell product shows high CAR expression levels in the CD3^+^CD56^+^, CD3^+^CD8^+^, and CD3^+^CD4^+^ cell subsets.

Emerging clinical evidence strongly supports the notion that the therapeutic efficacy of infused cells depends on the cell type, levels of CAR expression, dose of infused cells, and design of signaling moieties. In our cell type, the persistence of subsets with naive, effector, and stem cell memory [42, 43] (data not shown) phenotypes is indicative of a potential early and sustained response *in vivo*. Concerning CAR expression, cell numbers and effector activities, we demonstrated improved efficacy of our method when directly compared to available existing SB platforms applied to conventional T cells requiring repeated stimulations. [31, 32] An additional *in vivo* validation of our cell product in respect to already clinically proved CAR T-cell approaches may be of crucial interest before moving to human studies. With regard to signaling, in order to further increase specific proliferation, we performed combined CD28-OX40 costimulation, which has been previously shown to strengthen and sustain the activation signal and to reduce IL-10 secretion. [44, 45] Even though combined CD28-OX40 costimulation has been reported to stimulate CIK cells excessively, [46] we found that in immunodeficient mice CAR-redirected CIK cells were able to significantly decrease disease burden in previously established AML and ALL models. [27, 47]

Finally, contrary to viral vectors, we found a close-to-random distributions of SB integrations without preferences for gene dense regions and low tendency to integrate near gene promoters, as previously reported. [17, 19, 36] Most importantly, SB modified CIK cells did not show dominance of individual clones and selection of common insertion sites, which have been associated with potential leukemogenic insertional events in preclinical and clinical gene therapy. [10] Interestingly, we found a significant enrichment of genes expressed in T cells, probably caused by the temporal proximity of the modification and CD3-specific activation, since gene expression levels are known to influence SB intragenic integrations. [36] Furthermore, the loss of transposase expression during the third week of culture suggests that the remobilization of the transposon into deleterious loci should not occur.

In conclusion, here we provide evidence of a novel model of allogeneic non-viral gene transfer which represents a valid alternative to the use of viral vectors and patient-derived CAR T cells, currently restricted to only few specialized centers and a limited number of patients. Overall, the application of our method has the potential to address key clinical and manufacturing challenges in adoptive cell therapy for relapsed leukemic patients.

## MATERIALS AND METHODS

### Ethics statement

Investigation has been conducted in accordance with the ethical standards. The Institutional Review Board of the Ethical Committee of San Gerardo approved this study and informed consent has been obtained from patients or their guardians according to institutional guidelines and to the Helsinki Declaration.

### Cell lines and primary cells

All cell lines were maintained in culture with Advanced RPMI medium (Invitrogen, Carlsbad, CA) supplemented with 10% heat-inactivated FBS, 2mM L-glutamine, 25 IU/ml of penicillin and 25 mg/ml of streptomycin (Lonza, Basel, Switzerland). The THP-1 cell line was kindly provided by Dr. K. Fleischhauer, whereas REH, KG-1 and Nalm-6 cell lines were purchased directly from ATCC, where short tandem repeat analysis and cytogenetic studies are used to authenticate human cell lines. Primary cells from patients affected by AML and ALL were obtained from bone marrow (BM) and PB cells collected and frozen at diagnosis in San Gerardo Hospital (Table 1).

### Plasmids

The high-affinity human scFv for the CD123 antigen, generated starting from the DNA encoding mAb 7G3 [26, 48], was cloned in frame with CH_2_CH_3_-CD28-OX40-ζ from SFG-anti-CD33-CD28-OX40-ζ [49] as a transposon into a SB expression plasmid, pT-MNDU3-eGFP [23] replacing the eGFP sequence to obtain anti-CD123/pTMNDU3. The anti-CD19/pTMNDU3 was generated by replacing the scFvCD123 with the scFv from the SFG.aCD19 (clone FMC63) [50] kindly provided by Martin Pule, University College of London. The codon-optimized plasmids for SB transposase, pCMV-SB11, are described elsewhere. [47]

### CIK cell differentiation and modification

Human PBMCs, obtained from HDs upon informed consent, were isolated over Ficoll-Hypaque gradients (Pharmacia LKB, Uppsala, Sweden) and electroporated by 4D-Nucleofector^TM^ (Lonza) with 15 μg supercoiled DNA transposon plasmid coding for CARs or GFP (SB GFP, pT-MNDU3-eGFP) and 5 μg supercoiled DNA pCMV-SB11 plasmid coding for SB11 transposase using Amaxa™ 4D-Nucleofector™ EO-115 protocol (program 1) or EF-115 (program 2) and amaxa P3 Primary Cell 4D-Nucleofector kit (Lonza). As positive control of modification, the Amaxa GFP plasmid was employed according to the manufacturer's instruction. PBMCs, from the same source, irradiated with 60Gy of 137Cs γ-rays, were added to the samples previously electroporated in the presence of DNA. CIK-cell lines were differentiated by addition of IFN-γ (1000 U/ml; Dompè Biotec, Milano, Italy) at day 0 and of IL-2 (300 U/ml; Chiron B.V) and OKT-3 (50 ng/ml; Janssen-Cilag, Emeryville, CA) at day 1 as previously described. [25] Cells were then cultured for 21 days and fresh medium and IL-2 were added weekly. Subsequent analyses were performed on bulk CIK-cell lines.

### Flow cytometric analysis

T cells were tested for the expression of CD3 (clone SK7), CD8 (clone RPA-T8), CD4 (clone SK3), CD56 (clone B159), CD62L (clone DREG-56) and CD45RO (clone UCHL1) using specific antibodies (BD Bioscience, San Jose, CA), whereas leukemic blasts were assessed for CD45 (clone 2D1), CD33 (clone HIM3-4), CD123 (clone 7G3), CD19 (clone HIB19) expression (BD Bioscience) and CD10 (eBioCB-CALLA, eBioscience, San Diego, CA). For intracytoplasmic staining, T cells were stained with anti-CD3 mAb before fixation, permeabilization (Fixation/Permeabilization Solution Kit, BD Bioscience) and incubation with anti-human IFN-γ (B27) and IL-2 mAbs (MQ1-17H12, BD Pharmingen, San Diego, CA). CAR expression was detected using an anti-Human IgG (H+L) specific antibody (Jackson ImmunoResearch, Suffolk, UK), as previously described. [49] Samples were acquired using a BD FACS Canto flow cytometer (BD Biosciences), and data were analyzed with FlowJo 7.5.5 (Tree Star, Inc., Ashland, OR) and BD FACSDIVA™ (BD Biosciences). Quadrant markers were set accordingly to unstained controls.

### Cytotoxic assay

Cytotoxicity was evaluated in a 4-h co-culture assay at an effector:target (E:T) ratio of 5:1 by apoptosis detection with GFP-Certified™ Apoptosis/Necrosis detection kit (Enzo Life Sciences, Farmingdale, NY) and gating on target cells previously labeled with 5-(and 6)- Carboxyfluorescein diacetate succinimidyl ester, CFDA SE (CFSE, 1 μM, eBioscience). Briefly, killed cells was determined as percentage of Annexin V^+^Necrosis Detection Reagent (similar to 7-AAD)^−^ plus Annexin V^+^Necrosis Detection Reagent^+^ in CFSE^+^ target cells in co-culture with the effectors compared to target cells alone. Alternatively, flow cytometry-based quantitative analysis was employed to determine the percentage of viable target cells recovered from culture, stained with PE-anti-CD33/CFSE for AML target or PE-anti-CD19 for ALL cells. [26].

### CD107a/GZB mobilization assay

T-cell degranulation was evaluated in a CD107a flow cytometric assay. Briefly, 10^5^ CIKs were plated with anti-CD107a FITC mAb (4 μL/well; BD Pharmingen) in the presence or absence of 10^5^ target cells. After 3h, monensin A (Sigma-Aldrich, St Louis, MO) was added (30 μg/mL). After additional 3h of incubation, cells were washed and stained with anti-CD3, and anti-Human IgG (H+L) mAb.

### Cytokine detection

10^6^ T cells/ml were stimulated with leukemic blasts irradiated with 40Gy of 137Cs γ-rays at ratio of 1:1. After 48 h, culture supernatants were harvested and levels of cytokines were determined by ELISA according to the manufacturer's instruction (R&D Systems, Minneapolis, MN). The limit of detection was 15.6 pg/ml.

### Proliferation

10^6^ T cells/ml were stimulated with THP-1 and REH irradiated with 100 or 40Gy of 137Cs γ-rays, respectively, at 1:1 ratio. The ability of viable cells to cleave 3-(4,5-dimethylthiazol-2-yl)-2,5-diphenyltetrazolium bromide (MTT, Sigma-Aldrich) was measured according to the manufacturer's instruction. Alternatively, CIK-cell proliferation was determined by staining with 1 μM CFSE, as described elsewhere, [33] and CFSE dilution was analyzed by flow cytometry together with CAR expression and calculated by gating on CD3^+^ cells.

### Mice

Female 7-9 week NOD-SCID-γchain−/− (NSG) mice (The Jackson laboratory, Bar Harbor, ME) were transplanted with 5×10^6^ KG-1 or 1×10^6^ Nalm-6 cells using intravenous injection. Mice were then treated with 1×10^7^ CIK cells infused intravenously. All experiments were performed according to protocols approved by the Institutional Committees of Ministero della Salute and Milano-Bicocca University (N. 102/2013-B). All efforts were made to minimize the number of animals used and their suffering. For each experiment, we used groups of n ≥ 3 mice.

### Integration site retrieval and analysis

LAM-PCR was performed starting from 100ng of genomic DNA to collect integration sites, as previously described, [51] by using three restriction enzymes (HpyCH4IV, AciI and BfaI) and sequencing on an Illumina MiSeq sequencer, detailed in Supplementary Material.

### Quantitative Real-time PCR analysis for absolute detection of transposase enzyme

Total RNA was extracted with RNeasy Mini kit (Qiagen, Hilden, Germany), and cDNA was synthesized with SuperScript II Reverse Transcriptase in the presence of RNaseOUT Ribonuclease Inhibitor (Life Technologies, Carlsbad, CA) according to manufacturer's instructions. cDNA samples (25ng RNA equivalent) were run in duplicate or triplicate, and levels of transposase transcript were determined as relative expression by normalizing to GUS Control Gene Standards (Quiagen), detailed in Supplementary Material.

### Statistical analysis

Mean values were reported as Mean±SEM. Paired t test were used to determine the statistical significance of the data, with the exception of the *in vivo* experiments analyzed by the Mann-Whitney test. Two-tailed paired analysis was performed, unless otherwise specified in the text. Statistic calculations were performed with GraphPad Prism 5.0.

## SUPPLEMENTARY MATERIALS AND METHODS








