# Supplementary material for: Immunotherapy of acute leukemia by chimeric antigen receptor-modified lymphocytes using an improved Sleeping Beauty transposon platform
Source: Oncotarget. 2016 Jun 13;7(32):51581–97. doi: 10.18632/oncotarget.9955 (PMC5239498; doi:10.18632/oncotarget.9955)
Supplement: Supplementary file 1 [file oncotarget-07-51581-s001.pdf]

Immunotherapy of acute leukemia by chimeric antigen receptor-modified lymphocytes using an improved *Sleeping Beauty* transposon platform

MATERIALS AND METHODS

Conventional T-Cell differentiation and modification

PBMCs were electroporated by 4D-Nucleofector™ (Lonza) with 15 µg supercoiled DNA transposon plasmid coding for CARs (anti-CD19/pTMNDU3) and 5 µg supercoiled DNA pCMV-SB11 plasmid using Amaxa™ 4D-Nucleofector™ EO-115 protocol and amaxa P3 Primary Cell 4D-Nucleofector kit (Lonza). OKT3- and beads- activated T-cell lines were differentiated, as previously described, [1, 2] with and without addition of PBMCs irradiated, according to our method. OKT3-activated cells were then cultured for 21 days. CD19.CAR OKT3-activated cells in absence of irradiated PBMCs were then re-stimulated with rapid expansion protocol till day 30, as previously described, [2] since they expanded at lower extent compared to CIK cells. For the same reason, all beads-activated cell conditions were then re-stimulated with beads at day 14 till day 30, as previously described. [1] Accordingly, subsequent analyses were performed on bulk CIK and OKT3-activated cells at day 21 and on CD19.CAR OKT3-activated cells in absence of irradiated PBMCs and beads- activated cells at day 30.

Integration site retrieval and analysis

The LAM-PCR method starts with two steps of linear amplification using a 5'-biotynilated primer designed in forward direction on right IR/DR of the transposon under the following conditions: 95°C, for 5 min, 50 cycles at 95°C for 1 min, 60°C for 45 s, 72°C for 90 s, and a final step at 72°C for 10 min. After ligation o/n with streptavidin magnetic beads (Agencourt AMPure XP, Beckman Coulter Inc., Brea, CA) linear amplified products went through a hexanucleotide priming coupled to second-strand reconstitution, restriction enzyme digestion and ligation of a linker cassette. The biotinylated PCR product was denaturated, captured via magnetic beads, detached from beads and reamplified by two subsequent nested PCR with primers for right IRDR and linker cassette. We retrieved integration sites through the combination of LAM-PCR with the use of the restriction enzymes HpyCH4IV, AciI and BfaI. We then adapted the LAM-PCR products for sequencing on an Illumina MiSeq sequencer using the Illumina Truseq DNA Sample Preparation Kit LT (Illumina Inc., San Diego, CA).

Oligos used in the experiments are listed as follows (5' to 3'):

Linear amplification 5'Biotin-GCTTGTGGAAGG CTACTCGAAATGTTTGACCC

1st exponential PCR

Forward transposon 1: CCACTGGGAATGTGATG AAAGAAATAAAAGC

Reverse Linker cassette 1: GACCCGGGAGATCT GAATTC

2st exponential PCR

Forward transposon 2: AGACAGGGAATCTTTAC TCGGA

Reverse Linker cassette 2: GATCTGAATTCAGTGG CACAG

Sequence reads obtained from Illumina MiSeq platform were processed and mapped on the human genome (Hg19) with a previously described bioinformatics pipeline [3, 4] adapted to recognize SB-transposon-cellular genomic junctions.

Clonal abundance was estimated as the relative percentage of the number of sequencing reads representing each integration site with respect to the total of sequencing reads obtained.

For each integration site the pipeline identified the nearest gene and the resulting gene list used for subsequent analysis.

Quantitative Real-time PCR analysis for absolute detection of transposase enzyme

Levels of transposase transcript were quantified using Universal Probe Library System (Roche Diagnostic GmbH, Mannheim, Germany) with FastStart Universal Probe Master (Roche). Optimal primers and probe for transposase amplification were selected using Roche ProbeFinder software at Assay Design Center (https://www.roche-applied-science.com). In order to set up the standard curve, the copy number per µl was estimated according to the molecular weight of the vector and the insert. Six successive dilutions (from 10^7 to 10^1) were prepared and used to calculate the standard curve. Real time analysis was performed using 7900HT Fast Real-

Time PCR System platform (Life Technologies) with the following protocol: initial step at 95°C for 10 min, then 50 cycles at 95°C for 15 s and at 60°C for 30 s and by using SDS2.3 software. The corresponding standard curve generated a mean slope of -3.24 and an intercept of 37.43 Ct (cycle threshold). Data were reported using a threshold of 0.1. A mean Ct value of 24.36 was obtained for the 10^4 copies/ml dilution. Relative expression was determined by normalizing to GUS Control Gene Standards (Quiagen) expression in each set of samples to calculate a fold-change in value (standard curve with a mean slope of -3.38). The mean Ct value and the mean value of the log10 of the copy number for GUS control gene were used for the statistical analysis.

Oligos and probe used in the experiments are listed as follows (Probe Sequence 5' to 3'):

Probe # 87

Left primer AAGCCGAAGAACACCATCC

Right primer AGCACCCCCACAACATGA

PCR analysis for detection of TCR-Vβ rearrangements

Total DNA was extracted with QIAamp DNA Mini kit (Qiagen) according to manufacturer's instructions. PCR-based amplification of the TCR-Vβ gene rearrangements from the genomic DNA was carried out using specific primers in two reactions combining 23 Vβ primers and 13 Jβ primers, the first reaction with 23 Vβ primers and 9 Jβ primers covering JB1.1-1.6 and JB2.2, the second reaction with 23 Vβ primers and 4 Jβ primers covering JB2.1 and JB2.3-5 [5]. Detection of the PCR products was assessed by gel electrophoresis.

REFERENCES

1. Huang X, Guo H, Kang J, Choi S, Zhou TC, Tammana S, Lees CJ, Li ZZ, Milone M, Levine BL, Tolar J, June CH, Scott McIvor R, Wagner JE, Blazar BR and Zhou X. Sleeping Beauty transposon-mediated engineering of human primary T cells for therapy of CD19+ lymphoid malignancies. Mol Ther. 2008; 16:580-589.
2. Peng PD, Cohen CJ, Yang S, Hsu C, Jones S, Zhao Y, Zheng Z, Rosenberg SA and Morgan RA. Efficient nonviral Sleeping Beauty transposon-based TCR gene transfer to peripheral blood lymphocytes confers antigen-specific antitumor reactivity. Gene Ther. 2009; 16:1042-1049.
3. Biffi A, Montini E, Lorioli L, Cesani M, Fumagalli F, Plati T, Baldoli C, Martino S, Calabria A, Canale S, Benedicenti F, Vallanti G, Biasco L, Leo S, Kabbara N, Zanetti G, et al. Lentiviral hematopoietic stem cell gene therapy benefits metachromatic leukodystrophy. Science. 2013; 341:1233158.
4. Aiuti A, Biasco L, Scaramuzza S, Ferrua F, Cicalese MP, Baricordi C, Dionisio F, Calabria A, Giannelli S, Castiello MC, Bosticardo M, Evangelio C, Assanelli A, Casiraghi M, Di Nunzio S, Callegaro L, et al. Lentiviral hematopoietic stem cell gene therapy in patients with Wiskott-Aldrich syndrome. Science. 2013; 341:1233151.
5. van Dongen JJ, Langerak AW, Bruggemann M, Evans PA, Hummel M, Lavender FL, Delabesse E, Davi F, Schuuring E, Garcia-Sanz R, van Krieken JH, Droese J, Gonzalez D, Bastard C, White HE, Spaargaren M, et al. Design and standardization of PCR primers and protocols for detection of clonal immunoglobulin and T-cell receptor gene recombinations in suspect lymphoproliferations: report of the BIOMED-2 Concerted Action BMH4-CT98-3936. Leukemia. 2003; 17:2257-2317.

SUPPLEMENTARY FIGURES AND TABLES

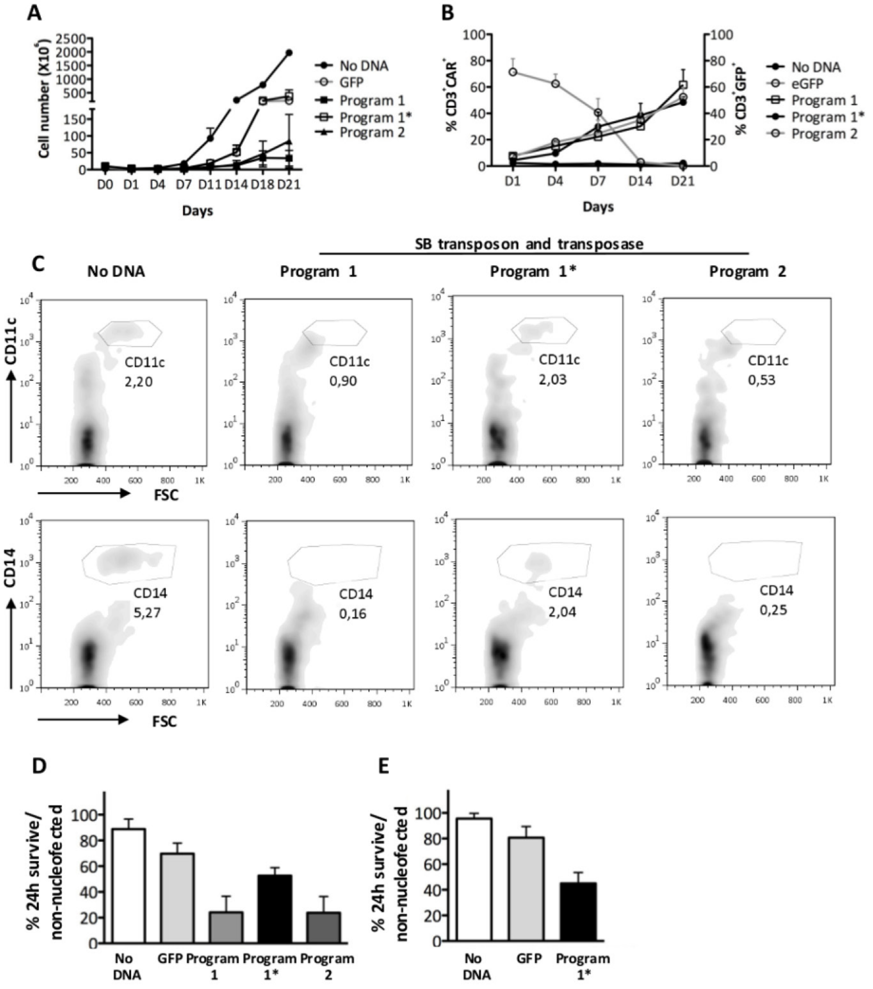

Supplementary Figure S1: Optimization of PBMCs modification by the SB system and CIK-cell expansion. **A.** Proliferation of cells nucleofected with program 1 in the absence of DNA, or with GFP, with transposase and transposon encoding CD123.CAR constructs in the absence or presence of simultaneous addition of γ-irradiated autologous PBMC (program 1 and program 1*, respectively) or, alternatively, with program 2 was followed overtime by cell count. Mean±SEM are relative to 3 donors. **B.** PBMCs modification was determined overtime by flow cytometric analysis of CD3 and CAR expression. **C.** The presence of CD11c$^+$ dendritic cells and of CD14$^+$ monocytes was determined by flow cytometric analysis at day 1 of differentiation. Representative flow cytometric results from one out of three tested donors are shown. Numbers represent the percentages of positive cells. **D.** Nucleofections were performed using the CD123.CAR construct, GFP control or no DNA. CD3$^+$ cell survival after 24h nucleofection was determined by cell count and normalized to non-nucleofected cells. Mean±SEM are relative to 3 donors for program 1 and 2, 10 for GFP, and 13 for no DNA and program 1*. **E.** Nucleofections were performed using the CD19.CAR construct, GFP control or No DNA. Mean±SEM are relative to 6 donors for No DNA, 5 for GFP, and 7 for program 1*.

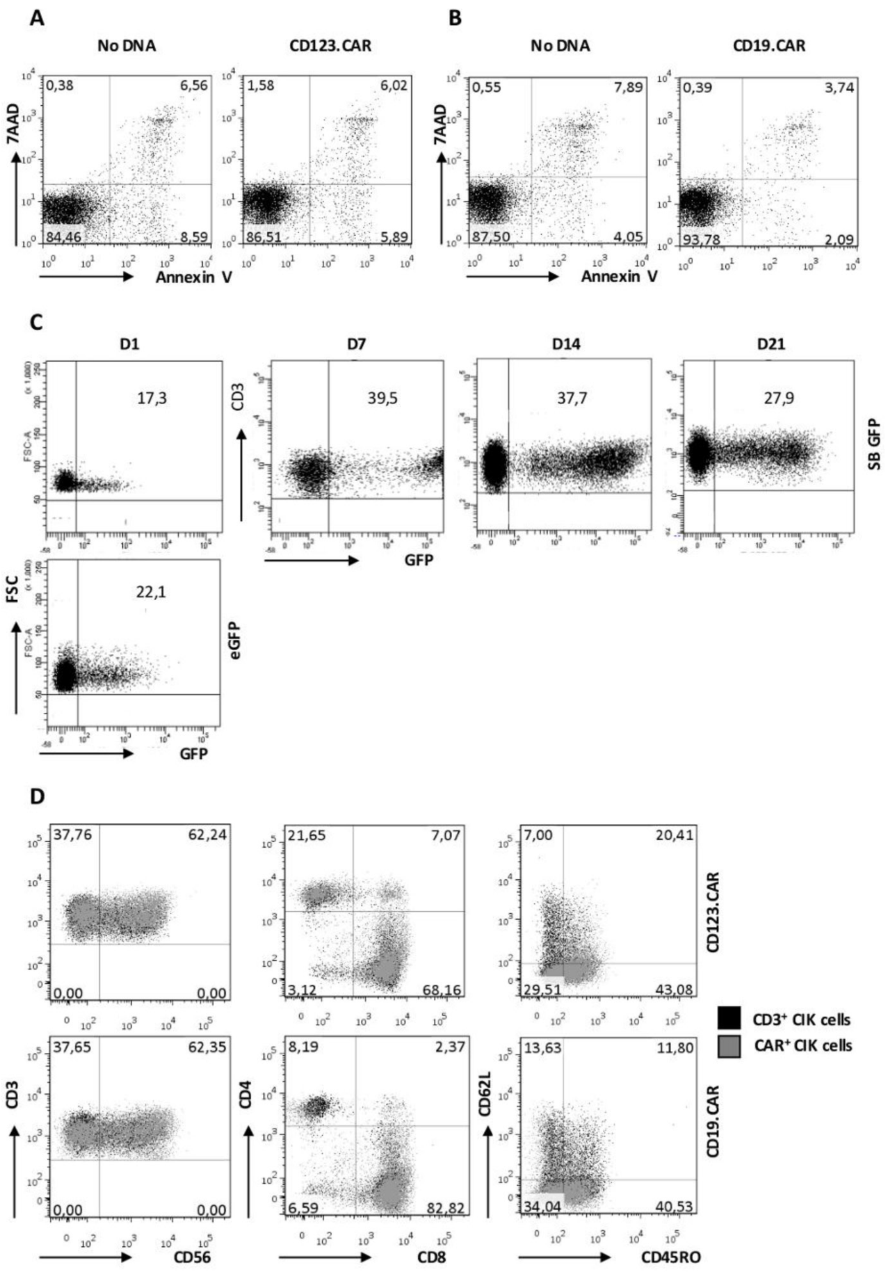

Supplementary Figure S2: Cell viability and CAR expression on T/CIK and memory subsets. A-B. Viability of CIK-cell cultures was determined as percentage of AnnexinV$^-$7-AAD$^-$ stained cells by flow cytometry. Representative flow cytometric results from one out of 13 donors tested for No DNA and CD123.CAR (A), and 8 donors tested for No DNA and CD19.CAR (B), are shown. Numbers represent the percentages of positive cells. **C.** Modification of cells nucleofected with GFP or with transposase and transposon encoding GFP was determined overtime by flow-cytometric analysis of FSC/CD3 and GFP expression.Representative results relative to one out of 4 donors are shown. Numbers represent the percentages of GFP positive cells. **D.** CAR expression in CD3$^+$CD56$^+$, CD3$^+$CD8$^+$/CD4$^+$, and CD3$^+$CD62L$^+$/CD45RO$^+$ cell populations was determined at day 21 of differentiation and overlaid as grey dot plot to total CD3$^+$ CIK-cell cultures (black dot plot). Results relative to one donor representative of 14 donors tested for CD123.CAR and 8 donors for CD19.CAR are shown. Numbers represent percentage of CAR$^+$ cells.

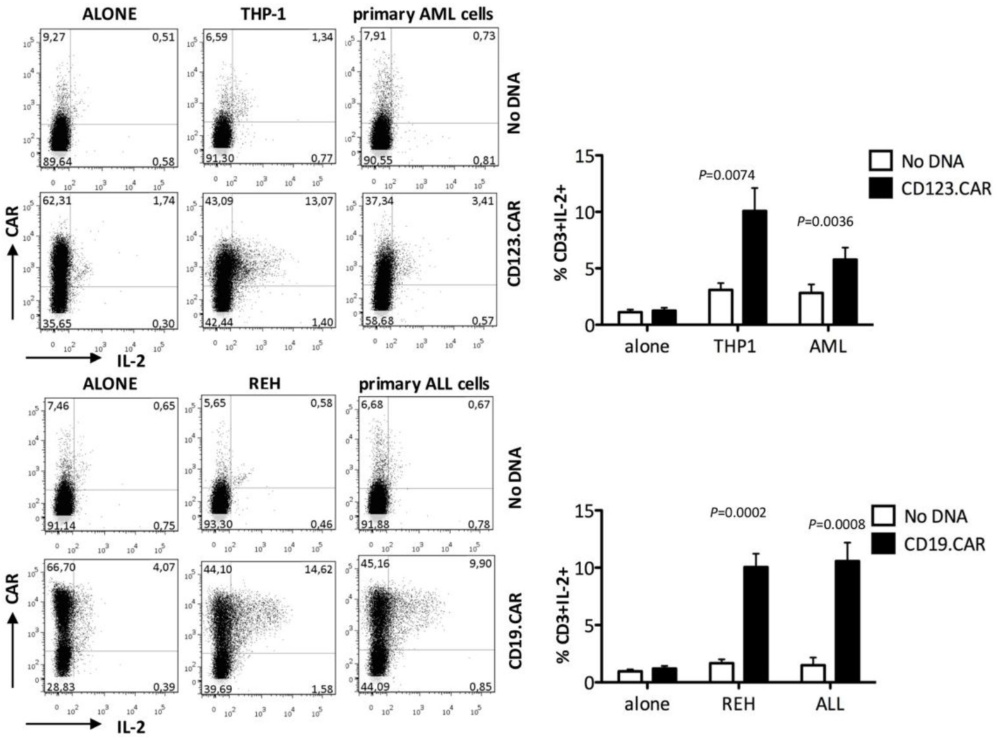

Supplementary Figure S3: Specific IL-2 production of CD123.CAR and CD19.CAR. IL-2 expression was determined in CD123.CAR and CD19.CAR cells by intracytoplasmic staining together with CAR surface staining upon stimulation with THP1 and REH, respectively, and against primary AML or ALL cells. No DNA cells were exposed to the same cell types as a negative control. Results relative to one donor representative of 9 donors for CD123.CAR and 8 donors for CD19.CAR are shown. Numbers represent the percentages of positive cells. Mean±SEM values are plotted alongside.

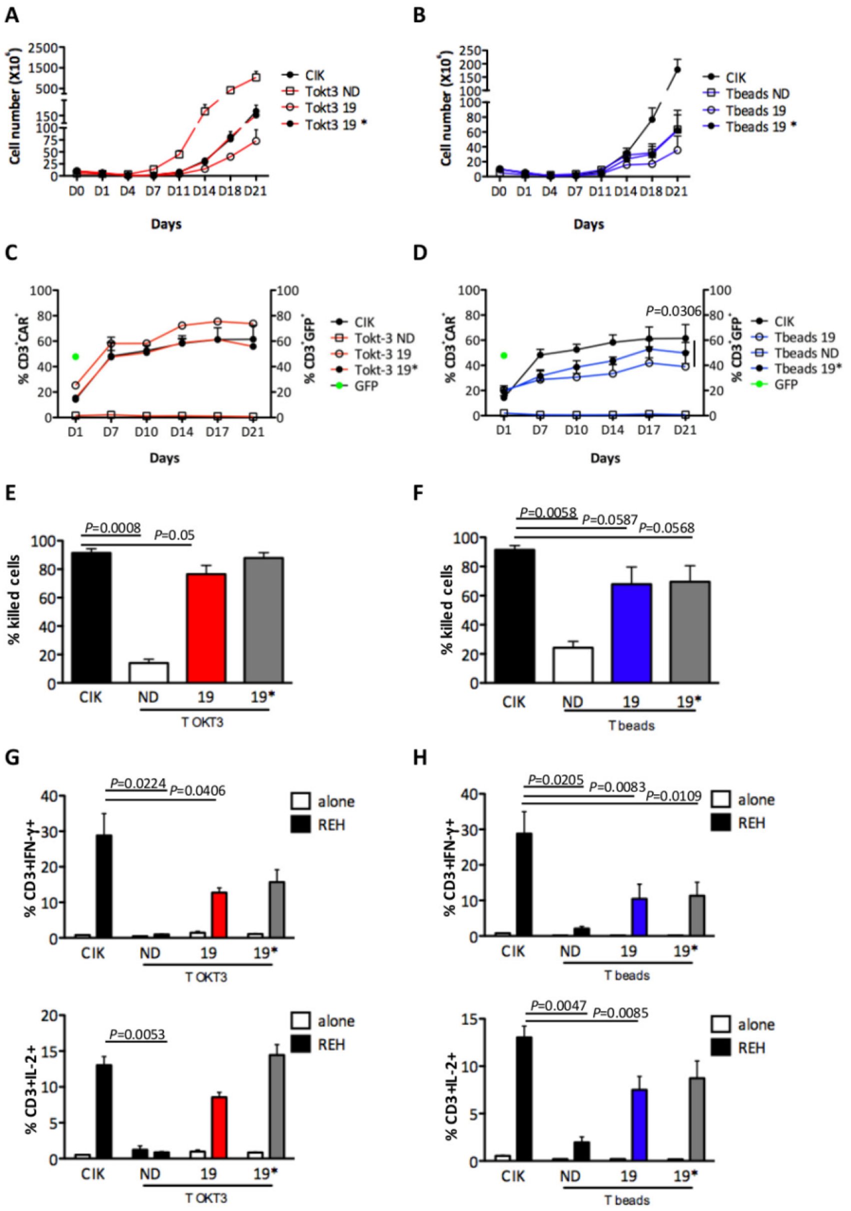

Supplementary Figure S4: Comparison of CIK-cell SB transposon platform with existing method. **A-B.** In comparison with CIK cells, proliferation of cells nucleofected in the absence of DNA (ND), with transposase and transposon encoding CD19.CAR construct in the absence or presence of simultaneous addition of γ-irradiated autologous PBMC (19 and 19*, respectively) stimulated as OKT3-activated (Tokt3, A) or beads-activated (Tbeads, B) conventional T cells, was followed overtime till day 21 by cell count. **C-D.** PBMCs modification was determined overtime till day 21 by flow-cytometric analysis of CD3 and CAR expression in Tokt3 (C) or Tbeads (D) cells. As positive control of modification, the Amaxa GFP plasmid was employed. **E-F.** Cytotoxic activity of modified Tokt3 (E) or Tbeads (F) cells against REH target cells was determined by apoptosis detection assay. The E:T ratio was 5:1. **G-H.** IFN-γ (upper panel) and IL-2 (lower panel) expression of modified Tokt3 (G) or Tbeads (H) cells was determined upon stimulation with REH by intracytoplasmic staining. P-values of the Paired t test (one-tailed) are indicated. Mean±SEM are relative to 3 donors.

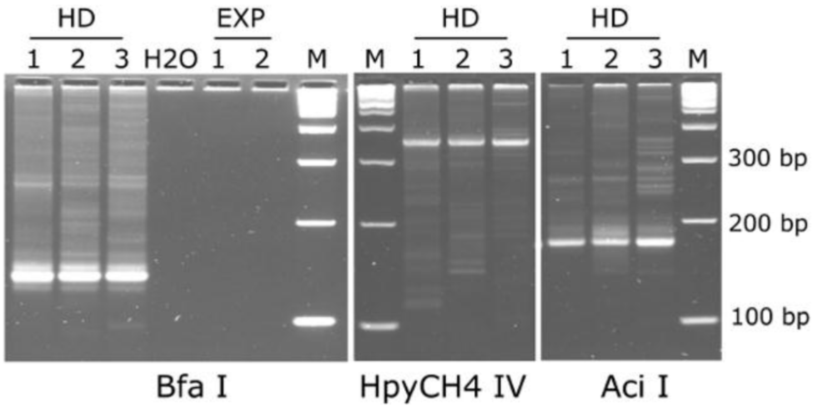

Supplementary Figure S5: Analysis of integration profile in engineered CIK cells. Spreadex gel electrophoresis of LAM-PCR products obtained from the genomic DNA of SB-marked CIK-cell cultures form 3 HD. The different restriction enzymes used for each amplification are indicated below each gel. H_2O, and EXP lanes are negative controls for the LAM-PCR steps of linear, first and second exponential amplifications respectively. M, molecular weight marker (fragment size are indicated in bp).

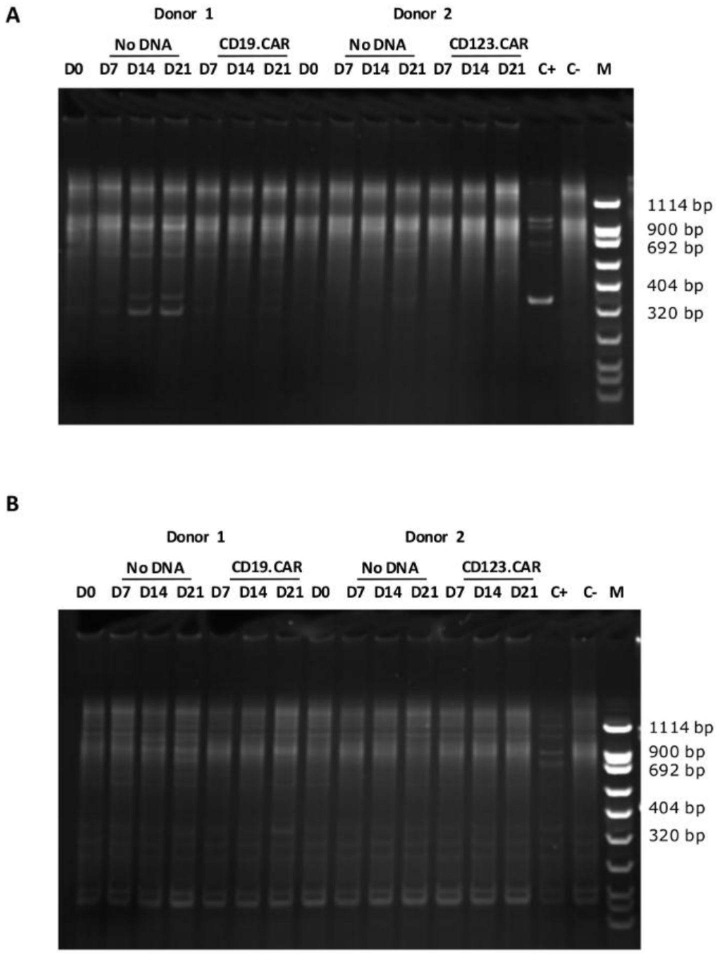

Supplementary Figure S6: TCR-Vβ of CIK cells modified by SB. Diffuse smears are present in CD123.CAR and CD19.CAR CIK cells at different time during differentiation, which reflects absence of detectable dominant TCR-Vβ gene rearrangements. **A.** PCR for the identification of the TCR-Vβ rearrangements (mix 1). **B.** PCR for the identification of the TCR-Vβ rearrangements (mix 2). Results from one representative donor out of 3 are shown.

Supplementary Table 1: Integration sites in engineered CIK cells.

See Supplementary File S1

Supplementary Table 2: Integration site abundance.

See Supplementary File S2

Supplementary Table 3: Common integration sites.

See Supplementary File S3
