# Supplementary material for: Immunotherapy of acute leukemia by chimeric antigen receptor-modified lymphocytes using an improved Sleeping Beauty transposon platform
Source: Oncotarget. 2016 Jun 13;7(32):51581–97. doi: 10.18632/oncotarget.9955 (PMC5239498; doi:10.18632/oncotarget.9955)
Supplement: Supplementary file 2 [file oncotarget-07-51581-s002.pdf]

HD1						
Chr	Integration locus	Integration strand	Gene symbol	Gene strand	Sequence count	% of reads
1	9504554	-	SPSB1	+	50	0,01362
1	21858529	-	ALPL	+	768	0,20913
1	26725462	+	LIN28A	+	6305	1,71691
1	26725678	-	LIN28A	+	1	0,00027
1	29088451	+	YTHDF2	+	518	0,14106
1	31506767	-	PUM1	-	26	0,00708
1	32721009	+	LCK	+	995	0,27095
1	33098558	+	ZBTB8OS	-	13	0,00354
1	36937914	+	CSF3R	-	518	0,14106
1	38148152	+	C1orf109	-	465	0,12662
1	39631435	+	MACF1	+	1	0,00027
1	40014728	+	PPIEL	-	12	0,00327
1	44395464	+	ST3GAL3	+	1302	0,35455
1	59589742	+	HSD52	-	4	0,00109
1	62975332	+	DOCK7	-	26	0,00708
1	78839706	+	MGC27382	+	120	0,03268
1	81538454	-	LPHN2	+	404	0,11001
1	86941142	-	CLCA1	+	9	0,00245
1	87502306	-	HS2ST1	+	4	0,00109
1	92361633	+	TGFBR3	-	890	0,24235
1	93026597	+	EVI5	-	14	0,00381
1	100795321	+	CDC14A	+	2121	0,57757
1	112096542	-	ADORA3	-	362	0,09858
1	149312596	+	LOC388692	+	1	0,00027
1	150125007	-	PLEKHO1	+	62	0,01688
1	151153421	-	VPS72	-	694	0,18898
1	151537034	+	TUFT1	+	442	0,12036
1	161218954	+	PCP4L1	+	1087	0,29600
1	161219109	+	PCP4L1	+	1	0,00027
1	166784357	-	POGK	+	1795	0,48879
1	169363473	-	C1orf114	-	10	0,00272
1	172995415	-	TNFSF18	-	112	0,03050
1	174985823	-	MRPS14	-	496	0,13507
1	178258605	+	RASAL2	+	867	0,23609
1	182845293	-	DHX9	+	761	0,20723
1	184072041	+	TSEN15	+	821	0,22357
1	186924443	+	PLA2G4A	+	1	0,00027
1	200053746	+	NR5A2	+	4	0,00109
1	201679597	+	NAV1	+	2280	0,62086
1	207620326	-	CR2	+	1608	0,43787
1	217583202	+	GPATCH2	-	500	0,13615
1	221659980	+	C1orf140	-	55	0,01498
1	222946901	+	FAM177B	+	592	0,16121
1	239701817	+	CHRM3	+	325	0,08850
1	243713076	-	AKT3	-	107	0,02914
2	11686694	+	GREB1	+	86	0,02342
2	15434547	-	NBAS	-	1340	0,36489
2	15699434	+	NBAS	-	287	0,07815
2	26085373	+	ASXL2	-	10244	2,78953
2	26328778	+	RAB10	+	7	0,00191
2	27819908	+	ZNF512	+	173	0,04711
2	28360579	-	BRE	+	47	0,01280
2	29441566	+	ALK	-	267	0,07271
2	30317700	+	YPEL5	+	127	0,03458
2	30548555	-	LBH	+	682	0,18571
2	44743064	+	MIR548AD	-	2116	0,57621
2	45778056	+	SRBD1	-	421	0,11464
2	54456879	+	TSPYL6	-	498	0,13561
2	60347663	-	MIR4432	-	1	0,00027
2	60347691	-	MIR4432	-	282	0,07679
2	69635541	+	NFU1	-	1	0,00027
2	71556329	+	ZNF638	+	886	0,24127
2	74498711	-	SLC4A5	-	19	0,00517
2	76238914	-	GCFC2	-	926	0,25216
2	82780886	-	LOC1720	+	17	0,00463
2	82784176	-	LOC1720	+	327	0,08905
2	96818050	+	DUSP2	-	4	0,00109

2	100002423	-	EIF5B	+	1	0,00027
2	102768613	+	IL1R1	+	283	0,07706
2	102888011	+	IL1RL2	+	117	0,03186
2	108871527	-	SULT1C3	+	161	0,04384
2	162853947	+	DPP4	-	816	0,22220
2	167825634	-	XIRP2	+	833	0,22683
2	172151750	-	METTL8	-	82	0,02233
2	174802802	+	SP3	-	621	0,16910
2	180319564	+	ZNF385B	-	2851	0,77635
2	181943049	-	UBE2E3	+	41	0,01116
2	191984508	-	STAT4	-	1	0,00027
2	197072655	-	HECW2	-	57	0,01552
2	197782744	-	PGAP1	-	1490	0,40574
2	201261288	-	SPATS2L	+	603	0,16420
2	225770698	+	DOCK10	-	295	0,08033
2	230898922	+	SLC16A14	-	2	0,00054
2	231643688	+	CAB39	+	153	0,04166
2	240608225	-	LOC150935	+	571	0,15549
3	9763146	+	CPNE9	+	219	0,05964
3	17487242	+	TBC1D5	-	179	0,04874
3	17709817	-	TBC1D5	-	31	0,00844
3	35468945	+	ARPP21	+	837	0,22792
3	37286776	-	GOLGA4	+	28	0,00762
3	38267043	-	OXSR1	+	881	0,23990
3	38283964	+	OXSR1	+	296	0,08060
3	46347477	-	CCR3	+	25	0,00681
3	47500937	+	SCAP	-	84	0,02287
3	50622378	-	HEMK1	+	4291	1,16848
3	51964071	-	RRP9	-	7090	1,93067
3	66345482	-	SLC25A26	+	1	0,00027
3	66345606	-	SLC25A26	+	7854	2,13871
3	72489140	+	RYBP	-	37	0,01008
3	99639304	-	FILIP1L	-	203	0,05528
3	108152827	-	MYH15	-	3	0,00082
3	115464212	-	GAP43	+	2967	0,80794
3	115915208	+	LSAMP	-	498	0,13561
3	119641863	-	GSK3B	-	404	0,11001
3	122985841	+	SEC22A	+	133	0,03622
3	126743681	+	PLXNA1	+	1	0,00027
3	127924873	+	EEFSEC	+	980	0,26686
3	127924902	+	EEFSEC	+	1	0,00027
3	129549646	+	TMCC1	-	16	0,00436
3	132565292	+	NPHP3-AS1	+	36	0,00980
3	137953935	-	ARMC8	+	43	0,01171
3	142637938	+	LOC100507389	+	3	0,00082
3	156747336	+	LEKR1	+	1	0,00027
3	163177677	-	LOC647107	-	1	0,00027
3	170568509	+	RPL22L1	-	166	0,04520
3	184575664	-	VPS8	+	243	0,06617
3	195747743	+	TFRC	-	8	0,00218
4	25379636	-	ANAPC4	+	275	0,07488
4	48834708	+	OCIAD1	+	1167	0,31778
4	53805492	-	SCFD2	-	261	0,07107
4	54727799	+	RPL21P44	-	2610	0,71073
4	57820422	-	NOA1	-	28	0,00762
4	61814084	-	LPHN3	+	235	0,06399
4	64941872	+	TECRL	-	488	0,13289
4	67293250	+	LOC100144602	+	2	0,00054
4	70610572	-	SULT1B1	-	149	0,04057
4	91170815	-	FAM190A	+	159	0,04330
4	99056323	-	C4orf37	-	60	0,01634
4	140204131	-	C4orf49	-	371	0,10103
4	146792342	+	ZNF827	-	105	0,02859
4	154663769	-	RNF175	-	395	0,10756
4	162479178	+	FSTL5	-	30	0,00817
4	169323031	-	DDX60L	-	2	0,00054
4	174075410	-	GALNT7	+	2	0,00054
4	175784727	+	GLRA3	-	655	0,17836
4	179879014	+	LOC285501	+	1	0,00027

5	6379268	+	MED10	-	1	0,00027
5	6379334	-	MED10	-	4579	1,24690
5	6569554	-	LOC255167	+	10	0,00272
5	21844511	+	CDH12	-	144	0,03921
5	39643745	+	DAB2	-	110	0,02995
5	55034831	-	DDX4	+	31	0,00844
5	61652440	+	KIF2A	+	1668	0,45421
5	63169724	-	HTR1A	-	1106	0,30117
5	77400377	+	AP3B1	-	1824	0,49669
5	79508954	-	SERINC5	-	1	0,00027
5	79509134	-	SERINC5	-	45	0,01225
5	92813273	+	FLJ42709	-	683	0,18599
5	95506170	+	MIR583	+	3270	0,89045
5	130222213	-	HINT1	-	10	0,00272
5	130755619	+	RAPGEF6	-	2045	0,55687
5	148607465	-	ABLIM3	+	15	0,00408
5	149728627	-	TCOF1	+	1	0,00027
5	156640064	-	ITK	+	88	0,02396
5	157255491	+	CLINT1	-	362	0,09858
5	157406052	+	CLINT1	-	16	0,00436
5	164222210	-	MAT2B	+	180	0,04902
5	169285150	+	FAM196B	-	587	0,15985
5	179951870	+	CNOT6	+	563	0,15331
5	179951907	+	CNOT6	+	1	0,00027
6	9146475	+	LOC100506207	+	1	0,00027
6	21641423	+	LINC00340	+	38	0,01035
6	25752428	+	SLC17A4	+	406	0,11056
6	31218134	-	HLA-C	-	1164	0,31697
6	35585597	+	FKBP5	-	2085	0,56776
6	38864462	+	DNAH8	+	500	0,13615
6	40749983	+	LRFN2	-	309	0,08414
6	42001202	-	CCND3	-	2	0,00054
6	55822900	-	BMP5	-	452	0,12308
6	57992706	-	GUSBP4	-	132	0,03594
6	65862730	-	EYS	-	2	0,00054
6	75547358	-	COL12A1	-	248	0,06753
6	81706824	+	BCKDHB	+	304	0,08278
6	96759697	-	FUT9	+	2	0,00054
6	107071202	+	RTN4IP1	-	2	0,00054
6	108868871	-	FOXO3	+	321	0,08741
6	120366208	-	LOC285762	-	962	0,26196
6	126164319	+	NCOA7	+	267	0,07271
6	139449363	-	HECA	+	1	0,00027
6	139449493	-	HECA	+	8110	2,20843
6	143195681	+	HIVEP2	-	168	0,04575
6	154054018	+	OPRM1	+	799	0,21757
6	158518109	+	SYNJ2	+	89	0,02424
7	1572637	+	MAFK	+	2037	0,55469
7	16980158	+	AGR3	-	181	0,04929
7	50115718	-	ZPBP	-	1024	0,27884
7	50297862	+	IKZF1	+	127	0,03458
7	50333086	-	IKZF1	+	32	0,00871
7	71433022	+	CALN1	-	852	0,23201
7	75293460	-	HIP1	-	1767	0,48117
7	77371133	-	RSBN1L	+	126	0,03431
7	85253446	-	SEMA3D	-	7	0,00191
7	85407759	-	SEMA3D	-	336	0,09150
7	100840533	-	MOGAT3	-	1	0,00027
7	105725968	+	SYPL1	-	733	0,19960
7	112344803	+	TMEM168	-	15	0,00408
7	112735161	+	GPR85	-	473	0,12880
7	127376637	-	SND1	+	1	0,00027
7	130447290	+	KLF14	-	447	0,12172
7	133949613	-	LRGUK	+	131	0,03567
7	149455956	-	ZNF467	-	142	0,03867
7	151161680	+	RHEB	-	316	0,08605
8	2108833	+	MYOM2	+	406	0,11056
8	5720949	+	LOC100287015	-	256	0,06971
8	12878500	-	KIAA1456	+	297	0,08088

8	28225470	+	ZNF395	-	152	0,04139
8	62113124	+	CLVS1	+	464	0,12635
8	68372089	-	CPA6	-	341	0,09286
8	70764527	-	SLCO5A1	-	482	0,13125
8	80558484	+	STMN2	+	22	0,00599
8	86184831	+	CA13	+	1415	0,38532
8	87169990	-	ATP6V0D2	+	6	0,00163
8	128067552	+	PCAT1	+	32	0,00871
8	129976078	+	LOC728724	-	299	0,08142
8	132867412	+	EFR3A	+	66	0,01797
8	134143736	-	TG	+	2	0,00054
8	143796453	-	LOC100288181	-	63	0,01716
9	5212811	-	INSL4	+	1	0,00027
9	31260458	-	LOC401497	-	35	0,00953
9	79277908	-	PRUNE2	-	18	0,00490
9	89786537	-	C9orf170	+	610	0,16611
9	96177945	+	FAM120AOS	-	7	0,00191
9	99026987	-	HSD17B3	-	11	0,00300
9	114163915	+	KIAA0368	-	21	0,00572
9	114555037	+	C9orf84	-	113	0,03077
9	115028050	-	PTBP3	-	2060	0,56096
9	115097155	+	PTBP3	-	9	0,00245
9	115338530	-	KIAA1958	+	205	0,05582
9	116188612	-	C9orf43	+	557	0,15168
9	121224378	-	DBC1	-	140	0,03812
9	126284999	+	DENND1A	-	1174	0,31969
9	129593603	+	ZBTB43	+	91	0,02478
9	131242489	-	ODF2	+	4136	1,12627
9	133578827	+	EXOSC2	+	298	0,08115
9	136965839	+	BRD3	-	7102	1,93394
10	14071668	+	FRMD4A	-	649	0,17673
10	16699302	+	RSU1	-	950	0,25869
10	19025291	+	ARL5B	+	246	0,06699
10	22202076	-	DNAJC1	-	2010	0,54734
10	34144025	-	LOC100505583	-	467	0,12717
10	52905179	+	PRKG1	+	8931	2,43199
10	54277526	-	DKK1	+	302	0,08224
10	73837933	-	SPOCK2	-	57	0,01552
10	73967866	+	ASCC1	-	28	0,00762
10	78892584	-	KCNMA1	-	18	0,00490
10	83255648	+	NRG3	+	1880	0,51194
10	84715539	-	NRG3	+	110	0,02995
10	85934808	+	C10orf99	+	1168	0,31806
10	90616020	-	ANKRD22	-	447	0,12172
10	90850452	+	MIR4679-2	-	43	0,01171
10	91224573	+	SLC16A12	-	1021	0,27803
10	101516210	+	CUTC	+	852	0,23201
10	102130548	-	LINC00263	+	83	0,02260
10	103349698	-	DPCD	+	7	0,00191
10	104333377	-	SUFU	+	241	0,06563
10	114464138	-	VTI1A	+	27	0,00735
10	120448820	+	C10orf46	-	430	0,11709
10	128478021	-	DOCK1	+	37	0,01008
10	135337025	-	CYP2E1	+	1272	0,34638
11	515872	+	RNH1	-	209	0,05691
11	5538486	-	UBQLNL	-	550	0,14977
11	9931734	-	SBF2	-	18	0,00490
11	22437542	+	SLC17A6	+	612	0,16665
11	34097384	-	CAPRIN1	+	731	0,19906
11	44140682	-	EXT2	+	244	0,06644
11	45182072	-	PRDM11	+	316	0,08605
11	54944827	+	TRIM48	+	2841	0,77363
11	58017821	-	OR10W1	-	465	0,12662
11	59405406	-	PATL1	-	1474	0,40138
11	63288930	-	LGALS12	+	22	0,00599
11	75618877	-	UVRAG	+	468	0,12744
11	86054046	+	C11orf73	+	244	0,06644
11	88075620	+	CTSC	-	1	0,00027
11	93821181	+	HEPHL1	+	567	0,15440

11	95683279	+	MTMR2	-	305	0,08305
11	95864700	-	MAML2	-	468	0,12744
11	95938154	-	MAML2	-	467	0,12717
11	101040981	-	PGR	-	4	0,00109
11	102247941	+	BIRC2	+	1258	0,34256
11	107586788	+	SLN	-	287	0,07815
11	108206559	-	ATM	+	125	0,03404
11	108218989	-	ATM	+	7	0,00191
11	109954311	+	ZC3H12C	+	1	0,00027
11	110096201	-	RDX	-	1	0,00027
11	118053057	+	SCN2B	-	104	0,02832
11	118136203	+	MPZL2	-	171	0,04656
11	118649272	+	DDX6	-	1700	0,46293
11	122686007	+	UBASH3B	+	1530	0,41663
11	125475290	-	STT3A	+	565	0,15385
11	128081151	-	ETS1	-	544	0,14814
11	134568500	+	LOC283177	+	75	0,02042
12	443992	+	KDM5A	-	1413	0,38477
12	721281	+	NINJ2	-	1	0,00027
12	7604863	-	CD163L1	-	1	0,00027
12	10537149	-	KLRK1	-	249	0,06780
12	10543107	-	KLRK1	-	103	0,02805
12	13305767	+	EMP1	+	196	0,05337
12	19276578	+	PLEKHA5	+	1267	0,34502
12	25803891	+	IFLTD1	-	3030	0,82510
12	47610163	+	FAM113B	+	7	0,00191
12	49652946	-	TUBA1C	+	2	0,00054
12	50142339	+	TMBIM6	+	93	0,02532
12	50170530	+	TMBIM6	+	116	0,03159
12	53595267	+	ITGB7	-	3	0,00082
12	54880812	-	NCKAP1L	+	37	0,01008
12	57020257	+	BAZ2A	-	5	0,00136
12	60045658	-	SLC16A7	+	405	0,11029
12	70690362	-	CNOT2	+	7	0,00191
12	70820222	+	KCNMB4	+	23	0,00626
12	77123860	+	ZDHHC17	+	1094	0,29791
12	85612225	+	LRRIQ1	+	283	0,07706
12	91795313	+	DCN	-	79	0,02151
12	99433725	-	ANKS1B	-	19	0,00517
12	104678265	+	TXNRD1	+	394	0,10729
12	110738780	-	ATP2A2	+	2231	0,60752
12	123522760	-	PITPNM2	-	776	0,21131
13	22004222	+	ZDHHC20	-	328	0,08932
13	30942321	-	LINC00426	-	1559	0,42453
13	34222559	-	STARD13	-	309	0,08414
13	42165576	+	KIAA0564	-	292	0,07951
13	43594683	-	DNAJC15	+	451	0,12281
13	45089285	-	TSC22D1	-	327	0,08905
13	46339133	-	SIAH3	-	314	0,08550
13	64270046	-	OR7E156P	+	661	0,18000
13	77382646	+	KCTD12	-	28	0,00762
13	90915917	+	MIR622	+	17062	4,64613
13	99999183	+	FKSG29	+	72	0,01961
13	101392113	+	TMTC4	-	636	0,17319
13	113871835	-	CUL4A	+	360	0,09803
14	20538241	+	OR4L1	+	8	0,00218
14	25025521	-	CTSG	-	1804	0,49125
14	33843865	-	NPAS3	+	436	0,11873
14	50173304	-	KLHDC1	+	98	0,02669
14	50351080	-	ARF6	+	94	0,02560
14	54886814	+	CDKN3	+	5	0,00136
14	56020400	+	KTN1-AS1	-	2	0,00054
14	56620913	-	PELI2	+	238	0,06481
14	62205149	+	HIF1A	+	41	0,01116
14	67818897	+	ATP6V1D	-	193	0,05256
14	69915531	-	SLC39A9	+	6	0,00163
14	74216887	-	MIR4505	+	721	0,19633
14	75954964	-	JDP2	+	1	0,00027
14	98832194	+	C14orf177	+	9	0,00245

14	104119634	-	KLC1	+	300	0,08169
14	104429595	+	TDRD9	+	859	0,23391
15	20019165	+	CHEK2P2	+	576	0,15685
15	20019605	-	CHEK2P2	+	1	0,00027
15	34444860	+	C15orf29	-	331	0,09013
15	43712986	+	TP53BP1	-	4247	1,15650
15	45135866	-	TRIM69	+	1	0,00027
15	50983220	+	TRPM7	-	961	0,26169
15	55125467	-	UNC13C	+	91	0,02478
15	60866543	-	RORA	-	4468	1,21668
15	65597300	+	PARP16	-	49	0,01334
15	71270754	-	LRRC49	+	43	0,01171
15	75007388	-	CYP1A1	-	1	0,00027
15	75007421	-	CYP1A1	-	736	0,20042
15	76909801	-	SCAPER	-	1	0,00027
15	78512501	+	ACSBG1	-	2519	0,68595
15	91209413	-	CRTC3	+	2197	0,59826
16	3373755	-	ZNF75A	+	1	0,00027
16	3768330	+	TRAP1	-	3683	1,00291
16	7043897	+	RBFOX1	+	306	0,08333
16	8766774	-	ABAT	+	188	0,05119
16	9176976	+	C16orf72	+	448	0,12199
16	11646328	+	LITAF	-	160	0,04357
16	11839652	+	TXNDC11	-	445	0,12118
16	21711471	-	OTOA	+	4119	1,12164
16	28302771	+	SBK1	+	54	0,01470
16	50094205	+	HEATR3	+	726	0,19770
16	50094294	-	HEATR3	+	1	0,00027
16	53770854	+	FTO	+	394	0,10729
16	58597838	-	CNOT1	-	497	0,13534
16	68255335	+	NFATC3	+	28	0,00762
16	69160515	-	CHTF8	-	177	0,04820
16	69914046	+	WWP2	+	6	0,00163
16	72970272	-	ZFHX3	-	25	0,00681
16	73333032	+	LOC100506172	+	196	0,05337
17	7467347	+	SENP3-EIF4A1	+	182	0,04956
17	8875222	+	PIK3R5	-	1	0,00027
17	13192214	-	HS3ST3A1	-	2	0,00054
17	14088125	-	COX10	+	4	0,00109
17	15484995	-	CDRT1	-	1401	0,38150
17	18668500	-	FBXW10	+	6	0,00163
17	19849526	-	AKAP10	-	27	0,00735
17	26410441	+	NLK	+	10	0,00272
17	33195806	-	CCT6B	-	320	0,08714
17	37963506	+	IKZF3	-	290	0,07897
17	38223212	-	THRA	+	47	0,01280
17	38709048	+	CCR7	-	615	0,16747
17	40616902	-	ATP6V0A1	+	40	0,01089
17	45848525	+	TBX21	+	4539	1,23601
17	50127399	-	CA10	-	179	0,04874
17	57513988	-	YPEL2	+	7	0,00191
17	57860815	+	VMP1	+	496	0,13507
17	60238464	-	MED13	-	320	0,08714
17	62566017	-	SMURF2	-	216	0,05882
17	62674317	+	SMURF2	-	378	0,10293
17	62674338	+	SMURF2	-	1	0,00027
17	62674352	+	SMURF2	-	1	0,00027
17	67082640	-	ABCA6	-	1	0,00027
17	69286553	+	SOX9	+	9319	2,53765
17	73340158	-	GRB2	-	649	0,17673
17	76159367	-	C17orf99	+	3739	1,01816
17	78488511	-	RPTOR	+	45	0,01225
17	78737761	-	RPTOR	+	1	0,00027
18	3093459	+	MYOM1	-	136	0,03703
18	4470661	-	DLGAP1	-	1447	0,39403
18	32914444	+	ZNF24	-	4	0,00109
18	38372096	-	KC6	-	1	0,00027
18	47334759	+	ACAA2	-	3	0,00082
18	50041623	-	DCC	+	2100	0,57185

18	54009119	+	LOC100505474	-	889	0,24208
18	55340267	+	ATP8B1	-	1569	0,42725
18	57633332	-	PMAIP1	+	212	0,05773
18	57879272	-	MC4R	-	3	0,00082
18	59017848	-	CDH20	+	154	0,04194
18	60887826	+	BCL2	-	348	0,09476
18	72627053	+	ZNF407	+	4874	1,32723
19	2098509	+	IZUMO4	+	1	0,00027
19	3334362	-	NFIC	+	5	0,00136
19	9818647	+	ZNF812	-	132	0,03594
19	12173541	+	ZNF844	+	55	0,01498
19	14637307	-	MIR639	+	17	0,00463
19	15119295	+	CCDC105	+	1	0,00027
19	15119470	-	CCDC105	+	1207	0,32868
19	15821851	-	CYP4F12	+	1463	0,39839
19	16273638	+	CIB3	-	78	0,02124
19	21835599	+	ZNF100	-	206	0,05610
19	21835663	+	ZNF100	-	1	0,00027
19	42422365	-	ARHGEF1	+	803	0,21866
19	42481899	+	ATP1A3	-	8757	2,38461
19	43018058	-	CEACAM1	-	11	0,00300
19	45810516	+	CKM	-	34	0,00926
19	49082439	-	SULT2B1	+	25	0,00681
19	52570512	+	ZNF841	-	210	0,05718
19	53941038	-	LOC147804	+	1326	0,36108
20	18102331	+	PET117	+	1032	0,28102
20	29619506	-	FRG1B	+	8	0,00218
20	31137636	-	LOC149950	+	298	0,08115
20	31628559	-	BPIFB6	+	1620	0,44114
20	43615509	+	STK4	+	14911	4,06040
20	51413714	-	TSHZ2	+	928	0,25270
20	52201341	-	ZNF217	-	1006	0,27394
21	33725813	-	URB1	-	71	0,01933
21	37629988	-	DOPEY2	+	1101	0,29981
21	37629996	-	DOPEY2	+	4	0,00109
21	38418447	-	PIGP	-	3866	1,05275
21	46219289	-	UBE2G2	-	43	0,01171
22	17646335	+	CECR5-AS1	+	271	0,07380
22	26185822	-	MYO18B	+	32	0,00871
22	29472413	-	KREMEN1	+	1	0,00027
22	31063260	-	DUSP18	-	4630	1,26079
22	40738431	+	ADSL	+	188	0,05119
22	40910812	+	MKL1	-	14616	3,98007
22	42588717	+	TCF20	-	1281	0,34883
22	45658074	+	KIAA0930	-	8548	2,32770
X	13383579	-	LOC100133123	+	56	0,01525
X	20243202	-	RPS6KA3	-	1748	0,47600
X	42037388	-	CASK	-	826	0,22493
X	46876802	-	PHF16	+	388	0,10566
X	47074130	-	UBA1	+	51	0,01389
X	54900595	-	TRO	+	19	0,00517
X	62522518	+	SPIN4	-	1	0,00027
X	71907770	-	PHKA1	-	17	0,00463
X	72779461	-	LOC139201	-	4	0,00109
X	86258507	+	DACH2	+	508	0,13833
X	118627441	-	SLC25A5	+	10	0,00272
X	122820480	-	THOC2	-	988	0,26904
X	123128903	+	STAG2	+	580	0,15794
X	123715395	+	ODZ1	-	174	0,04738
X	131590870	+	MBNL3	-	2	0,00054
X	152213080	-	PNMA3	+	938	0,25543

HD2

Chr	Integration locus	Integration strand	Gene symbol	Gene strand	Sequence count	% of reads
1	12080303	-	MIIP	+	3989	1,05760
1	17370071	+	SDHB	-	9659	2,56089
1	19293797	+	IFFO2	-	39124	10,37296
1	19293933	+	IFFO2	-	1	0,00027
1	25375656	-	RUNX3	-	2160	0,57268

1	26904146	-	RPS6KA1	+	1127	0,29880
1	27213782	-	GPN2	-	1421	0,37675
1	35564441	-	ZMYM1	+	1	0,00027
1	35564675	-	ZMYM1	+	1001	0,26540
1	36314559	-	EIF2C4	+	3544	0,93962
1	52691150	-	ZFYVE9	+	275	0,07291
1	86863636	-	ODF2L	-	968	0,25665
1	111679764	-	DRAM2	-	1	0,00027
1	145041972	-	PDE4DIP	-	93	0,02466
1	172195303	+	DNM3	+	1663	0,44091
1	172961624	+	TNFSF18	-	5	0,00133
1	181446932	+	CACNA1E	+	2604	0,69040
1	181447202	+	CACNA1E	+	1	0,00027
1	198762427	+	LOC100131234	-	5657	1,49984
1	198762620	+	LOC100131234	-	1	0,00027
1	199096051	+	LOC100131234	-	12	0,00318
1	203292079	-	BTG2	+	385	0,10208
1	211742777	+	SLC30A1	-	390	0,10340
1	235317768	-	RBM34	-	1	0,00027
2	29133389	+	WDR43	+	1002	0,26566
2	31017629	-	CAPN13	-	1864	0,49420
2	36223404	-	LOC100288911	-	1	0,00027
2	96968443	+	SNRNP200	-	15	0,00398
2	134862627	-	MIR3679	+	504	0,13363
2	136804573	-	DARS	-	5028	1,33308
2	158613542	-	ACVR1	-	86	0,02280
2	159800637	-	TANC1	+	1	0,00027
2	159800693	-	TANC1	+	2522	0,66866
2	162749655	+	SLC4A10	+	53	0,01405
2	172607265	+	DYNC1I2	+	9306	2,46730
2	191035649	-	C2orf88	+	2233	0,59204
2	201861608	-	FAM126B	-	2	0,00053
2	225873275	-	MIR4439	-	11	0,00292
3	1813628	+	CNTN4	+	1541	0,40857
3	15473875	+	EAF1	+	1056	0,27998
3	18686286	+	SATB1	-	193	0,05117
3	27126086	+	NEK10	-	1	0,00027
3	47846844	-	DHX30	+	1402	0,37171
3	52708851	+	PBRM1	-	688	0,18241
3	53827595	-	CACNA1D	+	11971	3,17388
3	59177654	+	C3orf67	-	3	0,00080
3	105874980	-	CBLB	-	24997	6,62746
3	112640249	+	CD200R1	-	3	0,00080
3	119714060	-	GSK3B	-	317	0,08405
3	149879897	+	LOC646903	+	128	0,03394
3	151789808	-	SUCNR1	+	5	0,00133
3	169966153	+	PRKCI	+	2274	0,60291
3	187903383	+	FLJ42393	+	8410	2,22975
3	189972943	-	CLDN1	-	2	0,00053
3	197516033	-	LRCH3	+	1	0,00027
3	197516039	-	LRCH3	+	2055	0,54484
4	457342	-	ZNF721	-	4095	1,08571
4	14980682	-	LOC441009	-	83	0,02201
4	40139130	-	N4BP2	+	3	0,00080
4	43190766	+	GRXCR1	+	218	0,05780
4	114369609	-	CAMK2D	-	232	0,06151
4	128090142	+	INTU	+	933	0,24737
4	147062935	+	LOC100505545	-	374	0,09916
4	183334218	-	ODZ3	+	892	0,23650
4	184620547	+	TRAPPC11	+	59	0,01564
5	7916353	-	MTRR	+	395	0,10473
5	12142253	-	CTNND2	-	1	0,00027
5	27758473	+	LOC643401	+	790	0,20945
5	54584902	-	DHX29	-	957	0,25373
5	64756328	+	ADAMTS6	-	16	0,00424
5	84260405	+	EDIL3	-	2	0,00053
5	91767870	-	FLJ42709	-	2179	0,57772
5	99956946	-	FAM174A	+	1	0,00027
5	99956986	-	FAM174A	+	44	0,01167

5	101829426	-	SLCO6A1	-	5491	1,45583
5	116565832	+	LOC728342	+	1020	0,27043
5	130701954	-	CDC42SE2	+	124	0,03288
5	138520445	-	SIL1	-	2	0,00053
5	165543731	-	ODZ2	+	299	0,07927
5	177736576	-	COL23A1	-	45	0,01193
6	34614037	-	C6orf106	-	17	0,00451
6	43032317	+	KLC4	+	5170	1,37072
6	88228870	+	RARS2	-	885	0,23464
6	127178541	-	RSPO3	+	1247	0,33062
6	156939286	+	ARID1B	+	434	0,11507
6	164021092	-	QKI	+	1078	0,28581
7	2440163	-	CHST12	+	1406	0,37277
7	13560724	-	ETV1	-	446	0,11825
7	14383530	-	DGKB	-	598	0,15855
7	36688352	+	AOAH	-	5225	1,38531
7	44660493	+	OGDH	+	3	0,00080
7	50506985	+	FIGNL1	-	1	0,00027
7	50507260	-	FIGNL1	-	3861	1,02367
7	67682868	-	STAG3L4	+	1515	0,40167
7	77172753	-	PTPN12	+	321	0,08511
7	80066872	+	GNAT3	-	838	0,22218
7	99206990	+	LOC100289187	+	3007	0,79725
7	100393768	-	ZAN	+	3	0,00080
7	102077129	-	ORAI2	+	281	0,07450
7	109468636	-	EIF3IP1	-	37	0,00981
7	133890685	+	LRGUK	+	791	0,20972
7	139778795	-	JHDM1D	-	122	0,03235
8	23094646	+	LOC389641	+	16	0,00424
8	59755566	+	TOX	-	1108	0,29376
8	81114555	-	TPD52	-	1	0,00027
8	91022225	-	DECR1	+	1013	0,26858
8	93039233	-	RUNX1T1	-	1	0,00027
8	102779476	+	NCALD	-	310	0,08219
8	116367115	+	TRPS1	-	569	0,15086
8	125125894	+	FER1L6	+	4473	1,18593
8	129228032	-	MIR1208	+	1835	0,48651
8	133331791	-	KCNQ3	-	523	0,13866
9	309198	-	DOCK8	+	182	0,04825
9	22591498	+	FLJ35282	+	1	0,00027
9	77783139	-	OSTF1	+	573	0,15192
9	92085353	+	SEMA4D	-	3	0,00080
9	135497488	+	DDX31	-	6519	1,72838
10	6652389	-	LOC439949	+	2348	0,62253
10	13491178	-	BEND7	-	14	0,00371
10	43935496	-	ZNF487P	+	280	0,07424
10	49981523	+	WDFY4	+	256	0,06787
10	75519293	+	SEC24C	+	361	0,09571
10	94033611	+	CPEB3	-	42	0,01114
10	121241611	-	RGS10	-	19	0,00504
10	125748194	+	CHST15	-	3798	1,00696
11	15050700	+	CALCB	+	2189	0,58037
11	27433051	-	LGR4	-	3	0,00080
11	31689318	-	ELP4	+	4	0,00106
11	47058940	-	C11orf49	+	124	0,03288
11	64475041	-	NRXN2	-	5176	1,37231
11	86750034	-	TMEM135	+	826	0,21900
11	111844231	-	DIXDC1	+	5054	1,33997
11	132486593	-	OPCML	-	998	0,26460
12	17899523	-	MIR3974	+	2	0,00053
12	42514402	-	GXYLT1	-	3745	0,99291
12	42774833	-	PPHLN1	+	5632	1,49321
12	50587332	-	LIMA1	-	26	0,00689
12	51373166	+	SLC11A2	-	730	0,19355
12	54439818	+	HOXC4	+	21	0,00557
12	56581610	+	SMARCC2	-	4919	1,30418
12	62998743	+	MIRLET7I	+	15881	4,21053
12	72868807	+	TRHDE	+	2	0,00053
12	74840052	-	ATXN7L3B	+	3	0,00080

12	91770183	+	DCN	-	1429	0,37887
12	93473698	+	LOC643339	-	4	0,00106
12	102955102	-	IGF1	-	10	0,00265
12	123358551	+	VPS37B	-	18	0,00477
12	127600584	+	LOC440117	-	146	0,03871
12	133488719	+	ZNF605	-	60	0,01591
13	26448242	-	ATP8A2	+	505	0,13389
13	45933680	+	TPT1-AS1	+	1	0,00027
13	49704688	-	FNDC3A	+	63	0,01670
13	52949052	-	THSD1	-	2	0,00053
13	82787320	-	SLITRK1	-	1	0,00027
13	95835270	-	ABCC4	-	5480	1,45291
14	23520708	-	CDH24	-	2792	0,74024
14	36281659	-	RALGAPA1	-	1	0,00027
14	36281754	-	RALGAPA1	-	6794	1,80130
14	39886256	-	FBXO33	-	7	0,00186
14	40184426	-	FBXO33	-	429	0,11374
14	75650300	+	TMED10	-	2111	0,55969
14	102289575	+	PPP2R5C	+	1987	0,52681
14	102831563	+	TECPR2	+	2651	0,70286
14	102831620	+	TECPR2	+	1	0,00027
15	40046912	-	FSIP1	-	4430	1,17453
15	45058736	+	TRIM69	+	5	0,00133
15	46573670	+	SQRDL	+	1047	0,27759
15	48588989	-	SLC12A1	+	2996	0,79433
15	50701781	-	USP8	+	8	0,00212
15	64833928	-	ZNF609	+	3	0,00080
15	65190549	-	ANKDD1A	+	94	0,02492
15	76226922	+	FBXO22	+	2046	0,54246
15	92448919	-	SLCO3A1	+	123	0,03261
16	56602613	-	MT4	+	416	0,11029
16	68887744	-	TMCO7	+	205	0,05435
16	71677248	-	MARVELD3	+	1260	0,33406
16	89828309	-	FANCA	-	1804	0,47830
17	459694	+	VPS53	-	1	0,00027
17	40310742	-	KCNH4	-	1	0,00027
17	47813879	+	FAM117A	-	221	0,05859
17	65490090	+	PITPNC1	+	24	0,00636
17	73696377	+	SAP30BP	+	4553	1,20714
17	73741559	-	ITGB4	+	3	0,00080
18	44781866	+	IER3IP1	-	248	0,06575
18	60793792	+	BCL2	-	2046	0,54246
18	64609920	+	CDH19	-	6640	1,76047
18	66791234	-	CCDC102B	+	32	0,00848
19	9361258	-	OR7E24	+	77	0,02042
19	10031126	+	OLFM2	-	25	0,00663
19	28101928	-	LOC148189	-	2178	0,57745
19	36185693	-	UPK1A	+	8196	2,17301
19	39154862	-	ACTN4	+	63	0,01670
19	51324932	-	KLK1	-	50	0,01326
19	53271057	+	ZNF600	-	1554	0,41201
19	54546645	+	VSTM1	-	17440	4,62387
20	7678349	+	HAO1	-	342	0,09067
20	8449963	+	PLCB1	+	64	0,01697
20	45687844	+	EYA2	+	3073	0,81475
21	17568748	-	LINC00478	+	8309	2,20297
21	42336388	+	DSCAM	-	319	0,08458
22	21326880	-	AIFM3	+	1461	0,38736
22	22594396	+	VPREB1	+	336	0,08908
22	27267108	-	MIAT	+	44	0,01167
22	29391482	+	ZNRF3	+	2125	0,56340
22	35549137	+	ISX	+	990	0,26248
22	42123124	-	MEI1	+	1	0,00027
X	52841815	+	XAGE5	+	5818	1,54253
X	54093379	+	FAM120C	-	1631	0,43243
X	78902769	-	ITM2A	-	4	0,00106
X	83505799	+	RPS6KA6	-	3393	0,89959
X	108978356	+	ACSL4	-	101	0,02678
X	150211117	-	HMGB3	+	2	0,00053

HD3						
Chr	Integration locus	Integration strand	Gene symbol	Gene strand	Sequence count	% of reads
1	10721430	-	CASZ1	-	1240	0,25030
1	32485255	+	KHDRBS1	+	2004	0,40452
1	32609636	+	KPNA6	+	7	0,00141
1	33199900	+	KIAA1522	+	640	0,12919
1	53027605	-	ZCCHC11	-	1	0,00020
1	78403034	-	NEXN	+	1429	0,28846
1	90142574	+	LRRC8C	+	145	0,02927
1	99224507	+	SNX7	+	1	0,00020
1	106600604	-	PRMT6	+	489	0,09871
1	111424904	-	CD53	+	24	0,00484
1	120520316	-	NOTCH2	-	105678	21,33198
1	147251917	-	GJA5	-	6	0,00121
1	151808704	-	C2CD4D	-	136	0,02745
1	154230727	-	UBAP2L	+	11	0,00222
1	155901703	+	KIAA0907	-	218	0,04401
1	172949086	-	TNFSF18	-	181	0,03654
1	173521494	+	SLC9A11	-	6787	1,37001
1	174666352	+	RABGAP1L	+	11	0,00222
1	198636980	-	PTPRC	+	113	0,02281
1	203048448	-	PPFIA4	+	275	0,05551
1	203862339	+	SNRPE	+	33	0,00666
1	225522829	+	DNAH14	+	476	0,09608
1	233340841	-	PCNXL2	-	1718	0,34679
1	244261740	+	ZNF238	+	1405	0,28361
2	10797756	-	NOL10	-	567	0,11445
2	11199977	+	FLJ33534	-	14	0,00283
2	26764687	+	OTOF	-	53	0,01070
2	32683198	-	BIRC6	+	30	0,00606
2	45646803	-	SRBD1	-	1035	0,20892
2	61193227	+	PUS10	-	2329	0,47013
2	61206788	+	PUS10	-	287	0,05793
2	70449707	-	TIA1	-	3879	0,78301
2	102394887	-	MAP4K4	+	8	0,00161
2	102817769	-	IL1RL2	+	12191	2,46085
2	109489830	-	CCDC138	+	251	0,05067
2	112410226	-	ANAPC1	-	3	0,00061
2	114568072	+	SLC35F5	-	325	0,06560
2	124069235	-	CNTNAP5	+	16	0,00323
2	127280658	-	GYPC	+	33	0,00666
2	135047042	+	MGAT5	+	621	0,12535
2	142104705	-	LRP1B	-	1074	0,21680
2	153957584	-	ARL6IP6	+	7	0,00141
2	162611220	-	SLC4A10	+	2	0,00040
2	167592445	+	XIRP2	+	65	0,01312
2	175420693	-	WIPF1	-	192	0,03876
2	191437144	-	TMEM194B	-	3284	0,66290
2	192023407	-	STAT4	-	3061	0,61789
2	197971761	-	ANKRD44	-	318	0,06419
2	200121648	-	SATB2	-	1	0,00020
2	207236725	+	ZDBF2	+	2848	0,57489
2	213967357	-	IKZF2	-	362	0,07307
2	219484004	-	PLCD4	+	17	0,00343
2	226581845	+	NYAP2	+	218	0,04401
2	237569109	-	CXCR7	+	2	0,00040
3	15355222	+	SH3BP5	-	741	0,14958
3	30342833	+	RBMS3	+	606	0,12233
3	48788481	-	PRKAR2A	-	4	0,00081
3	56988214	-	ARHGEF3	-	720	0,14534
3	63760849	+	C3orf49	+	1	0,00020
3	99395944	-	COL8A1	+	399	0,08054
3	107446646	-	BBX	+	8	0,00161
3	109080880	+	DPPA4	-	45	0,00908
3	115552976	+	LSAMP	-	2	0,00040
3	139645197	+	CLSTN2	+	1	0,00020
3	176897190	+	TBL1XR1	-	3	0,00061
3	184432002	+	MAGEF1	-	55	0,01110

4	10019114	-	SLC2A9	-	2	0,00040
4	10019171	-	SLC2A9	-	17638	3,56038
4	18004910	-	LCORL	-	179	0,03613
4	19907747	-	SLIT2	+	5081	1,02564
4	20926166	-	KCNIP4	-	5903	1,19157
4	26202765	+	RBPJ	+	4	0,00081
4	93433980	-	GRID2	+	322	0,06500
4	102224274	-	PPP3CA	-	1	0,00020
4	102224430	+	PPP3CA	-	39	0,00787
4	102224593	-	PPP3CA	-	7525	1,51898
4	109240414	+	LOC641518	+	35	0,00707
4	124114714	-	SPATA5	+	637	0,12858
4	126012855	+	FAT4	+	3914	0,79007
4	181810145	-	LINC00290	-	801	0,16169
4	185430562	+	IRF2	-	5	0,00101
5	10474449	-	ROPN1L	+	72	0,01453
5	33387573	-	TARS	+	1	0,00020
5	37708546	-	WDR70	+	173	0,03492
5	39873249	-	DAB2	-	747	0,15079
5	55676428	+	ANKRD55	-	8	0,00161
5	56850546	+	ACTBL2	-	471	0,09508
5	90733668	+	LOC100129716	+	2	0,00040
5	95149575	+	GLRX	-	473	0,09548
5	95472305	-	MIR583	+	172	0,03472
5	96476859	-	LIX1	-	153	0,03088
5	131958283	+	RAD50	+	677	0,13666
6	26050948	-	HIST1H3C	+	71	0,01433
6	43858275	-	LOC100132354	+	3	0,00061
6	86621353	-	SNHG5	-	57	0,01151
6	96560678	+	FUT9	+	35	0,00707
6	116987132	+	ZUFSP	-	1362	0,27493
6	119164889	+	MCM9	-	200	0,04037
6	130360190	+	L3MBTL3	+	401	0,08095
6	134286443	+	TBPL1	+	1144	0,23093
6	136990595	-	MAP3K5	-	6301	1,27191
6	142519252	-	VTA1	+	1	0,00020
6	143676542	+	AIG1	+	961	0,19399
6	153493478	+	RGS17	-	919	0,18551
6	155095380	+	SCAF8	+	5	0,00101
6	155693957	+	NOX3	-	251	0,05067
6	155878407	+	NOX3	-	1104	0,22285
6	157237693	-	ARID1B	+	465	0,09386
7	12123383	-	TMEM106B	+	4	0,00081
7	17986393	+	SNX13	-	103	0,02079
7	30550832	+	GGCT	-	950	0,19177
7	36651974	+	AOAH	-	10	0,00202
7	38217386	+	STARD3NL	+	7	0,00141
7	43722363	-	C7orf44	-	180	0,03633
7	44501572	-	NUDCD3	-	15	0,00303
7	81923069	+	CACNA2D1	-	1	0,00020
7	129488394	+	UBE2H	-	541	0,10921
8	17610279	-	MTUS1	-	1963	0,39625
8	19720459	+	INTS10	+	6	0,00121
8	20500062	-	LOC286114	+	1613	0,32560
8	30014475	+	DCTN6	+	760	0,15341
8	71489827	+	TRAM1	-	59	0,01191
8	78035874	+	PEX2	-	24	0,00484
8	87488099	+	FAM82B	-	683	0,13787
8	101268197	-	RNF19A	-	8	0,00161
8	106529732	-	ZFPM2	+	10420	2,10336
8	109288844	+	EIF3E	-	590	0,11910
8	124969062	+	FER1L6	+	2246	0,45337
8	127062871	+	LOC100130231	-	2350	0,47437
8	129108479	+	PVT1	+	158	0,03189
8	129843954	+	LOC728724	-	99	0,01998
9	72158532	-	APBA1	-	2083	0,42047
9	78795035	+	PCSK5	+	142	0,02866
9	82596925	+	TLE4	+	5	0,00101
9	91061239	-	SPIN1	+	3	0,00061

9	104474729	-	GRIN3A	-	292	0,05894
9	112675029	+	PALM2	+	5417	1,09347
9	114277245	+	ZNF483	+	1127	0,22749
9	133517723	+	FUBP3	+	630	0,12717
9	135271174	+	TTF1	-	4721	0,95297
10	4391238	+	LOC100216001	-	5831	1,17704
10	12697982	+	CAMK1D	+	340	0,06863
10	17039963	+	CUBN	-	2	0,00040
10	50856044	-	CHAT	+	4	0,00081
10	56954545	+	PCDH15	-	4	0,00081
10	75351780	+	USP54	-	1	0,00020
10	81092136	-	PPIF	+	6	0,00121
10	91439097	+	FLJ37201	-	440	0,08882
10	101121439	-	CNNM1	+	805	0,16250
10	104555631	+	C10orf26	+	67	0,01352
11	4391862	-	OR52B4	-	1651	0,33327
11	14059098	+	SPON1	+	102	0,02059
11	14981862	+	CALCA	-	19	0,00384
11	15855648	+	SOX6	-	252	0,05087
11	26043745	+	ANO3	+	161	0,03250
11	58355173	-	ZFP91-CNTF	+	43	0,00868
11	65292262	-	SCYL1	+	497	0,10032
11	66498618	-	SPTBN2	-	1459	0,29451
11	77942089	+	GAB2	-	44	0,00888
11	79976915	-	ODZ4	-	59	0,01191
11	96130261	+	JRKL	+	621	0,12535
11	113880579	+	HTR3A	+	765	0,15442
11	116930428	-	SIK3	-	1513	0,30541
11	120340340	+	ARHGEF12	+	8232	1,66170
11	123174063	+	MIR4493	-	1581	0,31914
12	8668503	+	CLEC4D	+	235	0,04744
12	11833457	-	ETV6	+	170	0,03432
12	19662278	+	AEBP2	+	994	0,20065
12	28492608	-	CCDC91	+	292	0,05894
12	45734365	-	ANO6	+	36	0,00727
12	47637227	+	FAM113B	+	1819	0,36718
12	48650854	-	OR10AD1	-	953	0,19237
12	50249314	-	FAIM2	-	31	0,00626
12	51471373	-	CSRNP2	-	1652	0,33347
12	53269504	+	KRT8	-	1	0,00020
12	65054941	+	RASSF3	+	377	0,07610
12	65569797	+	LEMD3	+	986	0,19903
12	72659594	+	LOC283392	-	402	0,08115
12	92729471	+	CLLU1OS	-	19	0,00384
12	93070210	-	C12orf74	+	2	0,00040
12	110852506	-	ANAPC7	-	971	0,19600
12	110852508	-	ANAPC7	-	1061	0,21417
12	116642466	+	MED13L	-	6	0,00121
12	120956759	-	COQ5	-	31	0,00626
12	121521931	-	OASL	-	18	0,00363
12	121558737	+	P2RX7	+	1	0,00020
13	40670899	+	LINC00548	-	20	0,00404
13	41232195	-	FOXO1	-	3287	0,66351
13	42646910	+	DGKH	+	131	0,02644
13	45959039	-	TPT1-AS1	+	557	0,11244
13	83509807	+	SLITRK1	-	504	0,10174
13	99896667	+	UBAC2	+	136	0,02745
13	109758672	-	MYO16	+	1530	0,30884
13	114315634	-	ATP4B	-	6	0,00121
13	115095522	+	CHAMP1	+	12	0,00242
14	20924631	+	APEX1	+	8889	1,79432
14	20947797	+	PNP	+	450	0,09084
14	23391592	+	PRMT5	-	6	0,00121
14	38003848	-	MIPOL1	+	192	0,03876
14	68093006	-	ARG2	+	6	0,00121
14	69230757	-	ZFP36L1	-	886	0,17885
14	77171905	+	VASH1	+	252	0,05087
14	77411096	-	C14orf166B	+	62	0,01252
14	77867857	-	NOXRED1	-	4	0,00081

14	97236788	+	VRK1	+	14	0,00283
14	98852825	+	C14orf177	+	2589	0,52261
14	99470390	+	BCL11B	-	1015	0,20489
15	31534514	-	LOC283710	-	1902	0,38393
15	31728086	+	OTUD7A	-	32	0,00646
15	33241491	+	FMN1	-	5486	1,10739
15	38876270	-	RASGRP1	-	971	0,19600
15	41983934	+	MIR626	+	4	0,00081
15	47913421	-	SEMA6D	+	581	0,11728
15	61140116	+	RORA	-	2331	0,47053
15	63857409	+	USP3	+	3917	0,79068
15	63916343	+	HERC1	-	353	0,07126
15	66055255	-	DENND4A	-	1921	0,38777
15	72563272	-	PARP6	-	1433	0,28926
15	76534768	-	ETFA	-	1	0,00020
15	78734744	-	IREB2	+	2	0,00040
15	82204534	-	MEX3B	-	1807	0,36476
15	91165492	+	CRTC3	+	12706	2,56481
15	100200088	-	MEF2A	+	43	0,00868
16	4581042	+	C16orf5	-	216	0,04360
16	11252244	-	CLEC16A	+	7	0,00141
16	23214084	-	SCNN1G	+	1152	0,23254
16	30741049	+	SRCAP	+	11	0,00222
16	55666332	-	SLC6A2	+	282	0,05692
16	69533066	-	CYB5B	+	533	0,10759
16	69654878	-	NFAT5	+	627	0,12657
16	74590808	-	GLG1	-	72	0,01453
17	4293681	-	UBE2G1	-	377	0,07610
17	6288551	+	AIPL1	-	11	0,00222
17	7497675	+	FXR2	-	845	0,17057
17	12062392	-	MAP2K4	+	37	0,00747
17	28462020	+	NSRP1	+	4	0,00081
17	38626528	+	TNS4	-	101	0,02039
17	40404128	-	STAT5B	-	57	0,01151
17	44265983	-	KIAA1267	-	16	0,00323
17	56811457	-	RAD51C	+	757	0,15281
17	72564296	+	CD300LD	-	1197	0,24162
17	75666741	+	LOC100507351	+	1675	0,33811
17	78905953	+	RPTOR	+	1749	0,35305
17	80215108	-	CSNK1D	-	478	0,09649
18	2721720	+	SMCHD1	+	5	0,00101
18	19338981	-	MIB1	+	9	0,00182
18	41728334	+	SETBP1	+	2220	0,44813
18	54993249	+	ST8SIA3	+	668	0,13484
18	59083023	-	CDH20	+	1110	0,22406
18	73057346	-	TSHZ1	+	75676	15,27583
18	74180638	-	ZNF516	-	1	0,00020
18	74180879	-	ZNF516	-	113	0,02281
19	1928093	+	SCAMP4	+	2	0,00040
19	4939350	-	UHRF1	+	1	0,00020
19	10140938	+	RDH8	+	1882	0,37990
19	10293219	+	DNMT1	-	41	0,00828
19	11713183	-	ZNF627	+	26	0,00525
19	12938107	+	RTBDN	-	103	0,02079
19	17377228	+	BABAM1	+	4	0,00081
19	42224064	+	CEACAM5	+	199	0,04017
19	45725314	+	EXOC3L2	-	3440	0,69439
19	53904033	-	ZNF765	+	17	0,00343
19	56385832	-	NLRP4	+	2	0,00040
19	56385939	-	NLRP4	+	6728	1,35810
19	56778748	+	ZSCAN5A	-	648	0,13080
19	58155158	-	ZNF211	+	522	0,10537
19	58954185	+	ZNF132	-	2637	0,53230
20	10779029	+	JAG1	-	76	0,01534
20	30937778	-	ASXL1	+	2	0,00040
20	31387788	-	DNMT3B	+	6	0,00121
20	47158873	+	PREX1	-	697	0,14070
20	47786722	+	STAU1	-	292	0,05894
20	47786815	-	STAU1	-	1159	0,23395

21	21270955	-	LINC00320	-	8276	1,67058
21	34500664	-	C21orf54	-	3218	0,64958
21	34600911	+	IFNAR2	+	5075	1,02443
21	35639450	-	LINC00310	+	1978	0,39928
21	44137254	-	PDE9A	+	253	0,05107
21	45462551	-	TRAPPC10	+	67	0,01352
21	46261904	+	PTTG1IP	-	58	0,01171
22	17956187	+	CECR2	+	37	0,00747
22	17956562	+	CECR2	+	1	0,00020
22	21947097	+	UBE2L3	+	18943	3,82380
22	21947132	-	UBE2L3	+	1	0,00020
22	21947163	-	UBE2L3	+	1	0,00020
22	37716297	+	CYTH4	+	10	0,00202
22	40738419	-	ADSL	+	1936	0,39080
X	50239789	+	DGKK	-	354	0,07146
X	53969628	+	PHF8	-	8	0,00161
X	64765371	+	FRMD8P1	-	2	0,00040
X	95523591	-	LOC643486	-	1529	0,30864
X	147223878	-	FMR1NB	+	1	0,00020
X	153161843	-	AVPR2	+	2334	0,47114
