# Supplementary material for: Immunotherapy of acute leukemia by chimeric antigen receptor-modified lymphocytes using an improved Sleeping Beauty transposon platform
Source: Oncotarget. 2016 Jun 13;7(32):51581–97. doi: 10.18632/oncotarget.9955 (PMC5239498; doi:10.18632/oncotarget.9955)
Supplement: Supplementary file 3 [file oncotarget-07-51581-s003.pdf]

HD1		
**Gene Symbol**	**Sequence Count**	**% of reads**
MIR622	17062	4,64613
STK4	14911	4,06040
MKL1	14616	3,98007
ASXL2	10244	2,78953
SOX9	9319	2,53765
PRKG1	8931	2,43199
ATP1A3	8757	2,38461
KIAA0930	8548	2,32770
HECA	8110	2,20843
SLC25A26	7854	2,13871
BRD3	7102	1,93394
RRP9	7090	1,93067
LIN28A	6305	1,71691
ZNF407	4874	1,32723
DUSP18	4630	1,26079
MED10	4579	1,24690
TBX21	4539	1,23601
RORA	4468	1,21668
HEMK1	4291	1,16848
TP53BP1	4247	1,15650
ODF2	4136	1,12627
OTOA	4119	1,12164
PIGP	3866	1,05275
C17orf99	3739	1,01816
TRAP1	3683	1,00291
All <1%	187210	50,97895
**Total reads**	**367230**	
**Unique IS**	**473**	

HD2		
**Gene Symbol**	**Sequence Count**	**% of reads**
IFFO2	39124	10,37296
CBLB	24997	6,62746
VSTM1	17440	4,62387
MIRLET7I	15881	4,21053
CACNA1D	11971	3,17388
SDHB	9659	2,56089
DYNC1I2	9306	2,46730
FLJ42393	8410	2,22975

LINC00478	8309	2,20297
UPK1A	8196	2,17301
RALGAPA1	6794	1,80130
CDH19	6640	1,76047
DDX31	6519	1,72838
XAGE5	5818	1,54253
LOC100131234	5657	1,49984
PPHLN1	5632	1,49321
SLCO6A1	5491	1,45583
ABCC4	5480	1,45291
AOAH	5225	1,38531
NRXN2	5176	1,37231
KLC4	5170	1,37072
DIXDC1	5054	1,33997
DARS	5028	1,33308
SMARCC2	4919	1,30418
SAP30BP	4553	1,20714
FER1L6	4473	1,18593
FSIP1	4430	1,17453
ZNF721	4095	1,08571
MIIP	3989	1,05760
FIGNL1	3861	1,02367
CHST15	3798	1,00696
All <1%	116078	30,77580
**Total reads**	**377173**	
**Unique IS**	**212**	

HD3		
**Gene Symbol**	**Sequence Count**	**% of reads**
NOTCH2	105678	21,33198
TSHZ1	75676	15,27583
UBE2L3	18943	3,82380
SLC2A9	17638	3,56038
CRTC3	12706	2,56481
IL1RL2	12191	2,46085
ZFPM2	10420	2,10336
APEX1	8889	1,79432
LINC00320	8276	1,67058
ARHGEF12	8232	1,66170
PPP3CA	7525	1,51898
SLC9A11	6787	1,37001

NLRP4	6728	1,35810
MAP3K5	6301	1,27191
KCNIP4	5903	1,19157
LOC100216001	5831	1,17704
FMN1	5486	1,10739
PALM2	5417	1,09347
SLIT2	5081	1,02564
IFNAR2	5075	1,02443
All <1%	156614	31,61384
**Total reads**	**495397**	
**Unique IS**	**293**	
