# Supplementary material for: Immunotherapy of acute leukemia by chimeric antigen receptor-modified lymphocytes using an improved Sleeping Beauty transposon platform
Source: Oncotarget. 2016 Jun 13;7(32):51581–97. doi: 10.18632/oncotarget.9955 (PMC5239498; doi:10.18632/oncotarget.9955)
Supplement: Supplementary file 4 [file oncotarget-07-51581-s004.pdf]

HD1		
Gene symbol	Highorder	Cluster
SMURF2	3	1
LIN28A	2	1
PCP4L1	2	2
MIR4432	2	1
LOC1720	2	2
OXSR1	2	1
SLC25A26	2	2
EEFSEC	2	3
MED10	2	1
SERINC5	2	2
CNOT6	2	3
HECA	2	1
ATM	2	1
KLRK1	2	1
TMBIM6	2	2
CHEK2P2	2	1
CYP1A1	2	2
HEATR3	2	1
CCDC105	2	1
ZNF100	2	2
DOPEY2	2	1
SPSB1	0	0
ALPL	0	0
YTHDF2	0	0
PUM1	0	0
LCK	0	0
ZBTB8OS	0	0
CSF3R	0	0
C1orf109	0	0
MACF1	0	0
PPIEL	0	0
ST3GAL3	0	0
HSD52	0	0
DOCK7	0	0
MGC27382	0	0
LPHN2	0	0
CLCA1	0	0

HS2ST1	0	0
TGFBR3	0	0
EVI5	0	0
CDC14A	0	0
ADORA3	0	0
LOC388692	0	0
PLEKHO1	0	0
VPS72	0	0
TUFT1	0	0
POGK	0	0
C1orf114	0	0
TNFSF18	0	0
MRPS14	0	0
RASAL2	0	0
DHX9	0	0
TSEN15	0	0
PLA2G4A	0	0
NR5A2	0	0
NAV1	0	0
CR2	0	0
GPATCH2	0	0
C1orf140	0	0
FAM177B	0	0
CHRM3	0	0
AKT3	0	0
GREB1	0	0
NBAS	0	0
ASXL2	0	0
RAB10	0	0
ZNF512	0	0
BRE	0	0
ALK	0	0
YPEL5	0	0
LBH	0	0
MIR548AD	0	0
SRBD1	0	0
TSPYL6	0	0
NFU1	0	0
ZNF638	0	0

SLC4A5	0	0
GCFC2	0	0
DUSP2	0	0
EIF5B	0	0
IL1R1	0	0
IL1RL2	0	0
SULT1C3	0	0
DPP4	0	0
XIRP2	0	0
METTL8	0	0
SP3	0	0
ZNF385B	0	0
UBE2E3	0	0
STAT4	0	0
HECW2	0	0
PGAP1	0	0
SPATS2L	0	0
DOCK10	0	0
SLC16A14	0	0
CAB39	0	0
LOC150935	0	0
CPNE9	0	0
TBC1D5	0	0
ARPP21	0	0
GOLGA4	0	0
CCR3	0	0
SCAP	0	0
HEMK1	0	0
RRP9	0	0
RYBP	0	0
FILIP1L	0	0
MYH15	0	0
GAP43	0	0
LSAMP	0	0
GSK3B	0	0
SEC22A	0	0
PLXNA1	0	0
TMCC1	0	0
NPHP3-AS1	0	0

ARMC8	0	0
LOC100507389	0	0
LEKR1	0	0
LOC647107	0	0
RPL22L1	0	0
VPS8	0	0
TFRC	0	0
ANAPC4	0	0
OCIAD1	0	0
SCFD2	0	0
RPL21P44	0	0
NOA1	0	0
LPHN3	0	0
TECRL	0	0
LOC100144602	0	0
SULT1B1	0	0
FAM190A	0	0
C4orf37	0	0
C4orf49	0	0
ZNF827	0	0
RNF175	0	0
FSTL5	0	0
DDX60L	0	0
GALNT7	0	0
GLRA3	0	0
LOC285501	0	0
LOC255167	0	0
CDH12	0	0
DAB2	0	0
DDX4	0	0
KIF2A	0	0
HTR1A	0	0
AP3B1	0	0
FLJ42709	0	0
MIR583	0	0
HINT1	0	0
RAPGEF6	0	0
ABLIM3	0	0
TCOF1	0	0

ITK	0	0
CLINT1	0	0
MAT2B	0	0
FAM196B	0	0
LOC100506207	0	0
LINC00340	0	0
SLC17A4	0	0
HLA-C	0	0
FKBP5	0	0
DNAH8	0	0
LRFN2	0	0
CCND3	0	0
BMP5	0	0
GUSBP4	0	0
EYS	0	0
COL12A1	0	0
BCKDHB	0	0
FUT9	0	0
RTN4IP1	0	0
FOXO3	0	0
LOC285762	0	0
NCOA7	0	0
HIVEP2	0	0
OPRM1	0	0
SYNJ2	0	0
MAFK	0	0
AGR3	0	0
ZPBP	0	0
IKZF1	0	0
CALN1	0	0
HIP1	0	0
RSBN1L	0	0
SEMA3D	0	0
MOGAT3	0	0
SYPL1	0	0
TMEM168	0	0
GPR85	0	0
SND1	0	0
KLF14	0	0

LRGUK	0	0
ZNF467	0	0
RHEB	0	0
MYOM2	0	0
LOC100287015	0	0
KIAA1456	0	0
ZNF395	0	0
CLVS1	0	0
CPA6	0	0
SLCO5A1	0	0
STMN2	0	0
CA13	0	0
ATP6V0D2	0	0
PCAT1	0	0
LOC728724	0	0
EFR3A	0	0
TG	0	0
LOC100288181	0	0
INSL4	0	0
LOC401497	0	0
PRUNE2	0	0
C9orf170	0	0
FAM120AOS	0	0
HSD17B3	0	0
KIAA0368	0	0
C9orf84	0	0
PTBP3	0	0
KIAA1958	0	0
C9orf43	0	0
DBC1	0	0
DENND1A	0	0
ZBTB43	0	0
ODF2	0	0
EXOSC2	0	0
BRD3	0	0
FRMD4A	0	0
RSU1	0	0
ARL5B	0	0
DNAJC1	0	0

LOC100505583	0	0
PRKG1	0	0
DKK1	0	0
SPOCK2	0	0
ASCC1	0	0
KCNMA1	0	0
NRG3	0	0
C10orf99	0	0
ANKRD22	0	0
MIR4679-2	0	0
SLC16A12	0	0
CUTC	0	0
LINC00263	0	0
DPCD	0	0
SUFU	0	0
VTI1A	0	0
C10orf46	0	0
DOCK1	0	0
CYP2E1	0	0
RNH1	0	0
UBQLNL	0	0
SBF2	0	0
SLC17A6	0	0
CAPRIN1	0	0
EXT2	0	0
PRDM11	0	0
TRIM48	0	0
OR10W1	0	0
PATL1	0	0
LGALS12	0	0
UVRAG	0	0
C11orf73	0	0
CTSC	0	0
HEPHL1	0	0
MTMR2	0	0
MAML2	0	0
PGR	0	0
BIRC2	0	0
SLN	0	0

ZC3H12C	0	0
RDX	0	0
SCN2B	0	0
MPZL2	0	0
DDX6	0	0
UBASH3B	0	0
STT3A	0	0
ETS1	0	0
LOC283177	0	0
KDM5A	0	0
NINJ2	0	0
CD163L1	0	0
EMP1	0	0
PLEKHA5	0	0
IFLTD1	0	0
FAM113B	0	0
TUBA1C	0	0
ITGB7	0	0
NCKAP1L	0	0
BAZ2A	0	0
SLC16A7	0	0
CNOT2	0	0
KCNMB4	0	0
ZDHHC17	0	0
LRRIQ1	0	0
DCN	0	0
ANKS1B	0	0
TXNRD1	0	0
ATP2A2	0	0
PITPNM2	0	0
ZDHHC20	0	0
LINC00426	0	0
STARD13	0	0
KIAA0564	0	0
DNAJC15	0	0
TSC22D1	0	0
SIAH3	0	0
OR7E156P	0	0
KCTD12	0	0

MIR622	0	0
FKSG29	0	0
TMTC4	0	0
CUL4A	0	0
OR4L1	0	0
CTSG	0	0
NPAS3	0	0
KLHDC1	0	0
ARF6	0	0
CDKN3	0	0
KTN1-AS1	0	0
PELI2	0	0
HIF1A	0	0
ATP6V1D	0	0
SLC39A9	0	0
MIR4505	0	0
JDP2	0	0
C14orf177	0	0
KLC1	0	0
TDRD9	0	0
C15orf29	0	0
TP53BP1	0	0
TRIM69	0	0
TRPM7	0	0
UNC13C	0	0
RORA	0	0
PARP16	0	0
LRRC49	0	0
SCAPER	0	0
ACSBG1	0	0
CRTC3	0	0
ZNF75A	0	0
TRAP1	0	0
RBFOX1	0	0
ABAT	0	0
C16orf72	0	0
LITAF	0	0
TXNDC11	0	0
OTOA	0	0

SBK1	0	0
FTO	0	0
CNOT1	0	0
NFATC3	0	0
CHTF8	0	0
WWP2	0	0
ZFHX3	0	0
LOC100506172	0	0
SENP3-EIF4A1	0	0
PIK3R5	0	0
HS3ST3A1	0	0
COX10	0	0
CDRT1	0	0
FBXW10	0	0
AKAP10	0	0
NLK	0	0
CCT6B	0	0
IKZF3	0	0
THRA	0	0
CCR7	0	0
ATP6V0A1	0	0
TBX21	0	0
CA10	0	0
YPEL2	0	0
VMP1	0	0
MED13	0	0
SMURF2	0	0
ABCA6	0	0
SOX9	0	0
GRB2	0	0
C17orf99	0	0
RPTOR	0	0
MYOM1	0	0
DLGAP1	0	0
ZNF24	0	0
KC6	0	0
ACAA2	0	0
DCC	0	0
LOC100505474	0	0

ATP8B1	0	0
PMAIP1	0	0
MC4R	0	0
CDH20	0	0
BCL2	0	0
ZNF407	0	0
IZUMO4	0	0
NFIC	0	0
ZNF812	0	0
ZNF844	0	0
MIR639	0	0
CYP4F12	0	0
CIB3	0	0
ARHGEF1	0	0
ATP1A3	0	0
CEACAM1	0	0
CKM	0	0
SULT2B1	0	0
ZNF841	0	0
LOC147804	0	0
PET117	0	0
FRG1B	0	0
LOC149950	0	0
BPIFB6	0	0
STK4	0	0
TSHZ2	0	0
ZNF217	0	0
URB1	0	0
PIGP	0	0
UBE2G2	0	0
CECR5-AS1	0	0
MYO18B	0	0
KREMEN1	0	0
DUSP18	0	0
ADSL	0	0
MKL1	0	0
TCF20	0	0
KIAA0930	0	0
LOC100133123	0	0

RPS6KA3	0	0
CASK	0	0
PHF16	0	0
UBA1	0	0
TRO	0	0
SPIN4	0	0
PHKA1	0	0
LOC139201	0	0
DACH2	0	0
SLC25A5	0	0
THOC2	0	0
STAG2	0	0
ODZ1	0	0
MBNL3	0	0
PNMA3	0	0

HD2		
Gene symbol	Highorder	Cluster
IFFO2	2	1
ZMYM1	2	2
CACNA1E	2	3
LOC100131234	2	4
TANC1	2	1
LRCH3	2	1
FAM174A	2	1
FIGNL1	2	1
RALGAPA1	2	1
TECPR2	2	2
MIIP	0	0
SDHB	0	0
RUNX3	0	0
RPS6KA1	0	0
GPN2	0	0
EIF2C4	0	0
ZFYVE9	0	0
ODF2L	0	0
DRAM2	0	0
PDE4DIP	0	0
DNM3	0	0

TNFSF18	0	0
LOC100131234	0	0
BTG2	0	0
SLC30A1	0	0
RBM34	0	0
WDR43	0	0
CAPN13	0	0
LOC100288911	0	0
SNRNP200	0	0
MIR3679	0	0
DARS	0	0
ACVR1	0	0
SLC4A10	0	0
DYNC1I2	0	0
C2orf88	0	0
FAM126B	0	0
MIR4439	0	0
CNTN4	0	0
EAF1	0	0
SATB1	0	0
NEK10	0	0
DHX30	0	0
PBRM1	0	0
CACNA1D	0	0
C3orf67	0	0
CBLB	0	0
CD200R1	0	0
GSK3B	0	0
LOC646903	0	0
SUCNR1	0	0
PRKCI	0	0
FLJ42393	0	0
CLDN1	0	0
ZNF721	0	0
LOC441009	0	0
N4BP2	0	0
GRXCR1	0	0
CAMK2D	0	0
INTU	0	0

LOC100505545	0	0
ODZ3	0	0
TRAPPC11	0	0
MTRR	0	0
CTNND2	0	0
LOC643401	0	0
DHX29	0	0
ADAMTS6	0	0
EDIL3	0	0
FLJ42709	0	0
SLCO6A1	0	0
LOC728342	0	0
CDC42SE2	0	0
SIL1	0	0
ODZ2	0	0
COL23A1	0	0
C6orf106	0	0
KLC4	0	0
RARS2	0	0
RSPO3	0	0
ARID1B	0	0
QKI	0	0
CHST12	0	0
ETV1	0	0
DGKB	0	0
AOAH	0	0
OGDH	0	0
STAG3L4	0	0
PTPN12	0	0
GNAT3	0	0
LOC100289187	0	0
ZAN	0	0
ORAI2	0	0
EIF3IP1	0	0
LRGUK	0	0
JHDM1D	0	0
LOC389641	0	0
TOX	0	0
TPD52	0	0

DECR1	0	0
RUNX1T1	0	0
NCALD	0	0
TRPS1	0	0
FER1L6	0	0
MIR1208	0	0
KCNQ3	0	0
DOCK8	0	0
FLJ35282	0	0
OSTF1	0	0
SEMA4D	0	0
DDX31	0	0
LOC439949	0	0
BEND7	0	0
ZNF487P	0	0
WDFY4	0	0
SEC24C	0	0
CPEB3	0	0
RGS10	0	0
CHST15	0	0
CALCB	0	0
LGR4	0	0
ELP4	0	0
C11orf49	0	0
NRXN2	0	0
TMEM135	0	0
DIXDC1	0	0
OPCML	0	0
MIR3974	0	0
GXYLT1	0	0
PPHLN1	0	0
LIMA1	0	0
SLC11A2	0	0
HOXC4	0	0
SMARCC2	0	0
MIRLET7I	0	0
TRHDE	0	0
ATXN7L3B	0	0
DCN	0	0

LOC643339	0	0
IGF1	0	0
VPS37B	0	0
LOC440117	0	0
ZNF605	0	0
ATP8A2	0	0
TPT1-AS1	0	0
FNDC3A	0	0
THSD1	0	0
SLITRK1	0	0
ABCC4	0	0
CDH24	0	0
FBXO33	0	0
TMED10	0	0
PPP2R5C	0	0
FSIP1	0	0
TRIM69	0	0
SQRDL	0	0
SLC12A1	0	0
USP8	0	0
ZNF609	0	0
ANKDD1A	0	0
FBXO22	0	0
SLCO3A1	0	0
MT4	0	0
TMCO7	0	0
MARVELD3	0	0
FANCA	0	0
VPS53	0	0
KCNH4	0	0
FAM117A	0	0
PITPNC1	0	0
SAP30BP	0	0
ITGB4	0	0
IER3IP1	0	0
BCL2	0	0
CDH19	0	0
CCDC102B	0	0
OR7E24	0	0

OLFM2	0	0
LOC148189	0	0
UPK1A	0	0
ACTN4	0	0
KLK1	0	0
ZNF600	0	0
VSTM1	0	0
HAO1	0	0
PLCB1	0	0
EYA2	0	0
LINC00478	0	0
DSCAM	0	0
AIFM3	0	0
VPREB1	0	0
MIAT	0	0
ZNRF3	0	0
ISX	0	0
MEI1	0	0
XAGE5	0	0
FAM120C	0	0
ITM2A	0	0
RPS6KA6	0	0
ACSL4	0	0
HMGB3	0	0

HD3		
Gene symbol	Highorder	Cluster
PPP3CA	3	2
UBE2L3	3	2
PUS10	2	1
SLC2A9	2	1
ANAPC7	2	1
APEX1	2	1
PNP	2	1
ZNF516	2	1
NLRP4	2	1
STAU1	2	1
CECR2	2	1
CASZ1	0	0

KHDRBS1	0	0
KPNA6	0	0
KIAA1522	0	0
ZCCHC11	0	0
NEXN	0	0
LRRC8C	0	0
SNX7	0	0
PRMT6	0	0
CD53	0	0
NOTCH2	0	0
GJA5	0	0
C2CD4D	0	0
UBAP2L	0	0
KIAA0907	0	0
TNFSF18	0	0
SLC9A11	0	0
RABGAP1L	0	0
PTPRC	0	0
PPFIA4	0	0
SNRPE	0	0
DNAH14	0	0
PCNXL2	0	0
ZNF238	0	0
NOL10	0	0
FLJ33534	0	0
OTOF	0	0
BIRC6	0	0
SRBD1	0	0
TIA1	0	0
MAP4K4	0	0
IL1RL2	0	0
CCDC138	0	0
ANAPC1	0	0
SLC35F5	0	0
CNTNAP5	0	0
GYPC	0	0
MGAT5	0	0
LRP1B	0	0
ARL6IP6	0	0

SLC4A10	0	0
XIRP2	0	0
WIPF1	0	0
TMEM194B	0	0
STAT4	0	0
ANKRD44	0	0
SATB2	0	0
ZDBF2	0	0
IKZF2	0	0
PLCD4	0	0
NYAP2	0	0
CXCR7	0	0
SH3BP5	0	0
RBMS3	0	0
PRKAR2A	0	0
ARHGEF3	0	0
C3orf49	0	0
COL8A1	0	0
BBX	0	0
DPPA4	0	0
LSAMP	0	0
CLSTN2	0	0
TBL1XR1	0	0
MAGEF1	0	0
LCORL	0	0
SLIT2	0	0
KCNIP4	0	0
RBPJ	0	0
GRID2	0	0
LOC641518	0	0
SPATA5	0	0
FAT4	0	0
LINC00290	0	0
IRF2	0	0
ROPN1L	0	0
TARS	0	0
WDR70	0	0
DAB2	0	0
ANKRD55	0	0

ACTBL2	0	0
LOC100129716	0	0
GLRX	0	0
MIR583	0	0
LIX1	0	0
RAD50	0	0
HIST1H3C	0	0
LOC100132354	0	0
SNHG5	0	0
FUT9	0	0
ZUFSP	0	0
MCM9	0	0
L3MBTL3	0	0
TBPL1	0	0
MAP3K5	0	0
VTA1	0	0
AIG1	0	0
RGS17	0	0
SCAF8	0	0
NOX3	0	0
ARID1B	0	0
TMEM106B	0	0
SNX13	0	0
GGCT	0	0
AOAH	0	0
STARD3NL	0	0
C7orf44	0	0
NUDCD3	0	0
CACNA2D1	0	0
UBE2H	0	0
MTUS1	0	0
INTS10	0	0
LOC286114	0	0
DCTN6	0	0
TRAM1	0	0
PEX2	0	0
FAM82B	0	0
RNF19A	0	0
ZFPM2	0	0

EIF3E	0	0
FER1L6	0	0
LOC100130231	0	0
PVT1	0	0
LOC728724	0	0
APBA1	0	0
PCSK5	0	0
TLE4	0	0
SPIN1	0	0
GRIN3A	0	0
PALM2	0	0
ZNF483	0	0
FUBP3	0	0
TTF1	0	0
LOC100216001	0	0
CAMK1D	0	0
CUBN	0	0
CHAT	0	0
PCDH15	0	0
USP54	0	0
PPIF	0	0
FLJ37201	0	0
CNNM1	0	0
C10orf26	0	0
OR52B4	0	0
SPON1	0	0
CALCA	0	0
SOX6	0	0
ANO3	0	0
ZFP91-CNTF	0	0
SCYL1	0	0
SPTBN2	0	0
GAB2	0	0
ODZ4	0	0
JRKL	0	0
HTR3A	0	0
SIK3	0	0
ARHGEF12	0	0
MIR4493	0	0

CLEC4D	0	0
ETV6	0	0
AEBP2	0	0
CCDC91	0	0
ANO6	0	0
FAM113B	0	0
OR10AD1	0	0
FAIM2	0	0
CSRNP2	0	0
KRT8	0	0
RASSF3	0	0
LEMD3	0	0
LOC283392	0	0
CLLU1OS	0	0
C12orf74	0	0
MED13L	0	0
COQ5	0	0
OASL	0	0
P2RX7	0	0
LINC00548	0	0
FOXO1	0	0
DGKH	0	0
TPT1-AS1	0	0
SLITRK1	0	0
UBAC2	0	0
MYO16	0	0
ATP4B	0	0
CHAMP1	0	0
PRMT5	0	0
MIPOL1	0	0
ARG2	0	0
ZFP36L1	0	0
VASH1	0	0
C14orf166B	0	0
NOXRED1	0	0
VRK1	0	0
C14orf177	0	0
BCL11B	0	0
LOC283710	0	0

OTUD7A	0	0
FMN1	0	0
RASGRP1	0	0
MIR626	0	0
SEMA6D	0	0
RORA	0	0
USP3	0	0
HERC1	0	0
DENND4A	0	0
PARP6	0	0
ETFA	0	0
IREB2	0	0
MEX3B	0	0
CRTC3	0	0
MEF2A	0	0
C16orf5	0	0
CLEC16A	0	0
SCNN1G	0	0
SRCAP	0	0
SLC6A2	0	0
CYB5B	0	0
NFAT5	0	0
GLG1	0	0
UBE2G1	0	0
AIPL1	0	0
FXR2	0	0
MAP2K4	0	0
NSRP1	0	0
TNS4	0	0
STAT5B	0	0
KIAA1267	0	0
RAD51C	0	0
CD300LD	0	0
LOC100507351	0	0
RPTOR	0	0
CSNK1D	0	0
SMCHD1	0	0
MIB1	0	0
SETBP1	0	0

ST8SIA3	0	0
CDH20	0	0
TSHZ1	0	0
SCAMP4	0	0
UHRF1	0	0
RDH8	0	0
DNMT1	0	0
ZNF627	0	0
RTBDN	0	0
BABAM1	0	0
CEACAM5	0	0
EXOC3L2	0	0
ZNF765	0	0
ZSCAN5A	0	0
ZNF211	0	0
ZNF132	0	0
JAG1	0	0
ASXL1	0	0
DNMT3B	0	0
PREX1	0	0
LINC00320	0	0
C21orf54	0	0
IFNAR2	0	0
LINC00310	0	0
PDE9A	0	0
TRAPPC10	0	0
PTTG1IP	0	0
CYTH4	0	0
ADSL	0	0
DGKK	0	0
PHF8	0	0
FRMD8P1	0	0
LOC643486	0	0
FMR1NB	0	0
AVPR2	0	0
